# Selenoproteins synergistically protect porcine skeletal muscle from oxidative damage via relieving mitochondrial dysfunction and endoplasmic reticulum stress

**DOI:** 10.1186/s40104-023-00877-6

**Published:** 2023-06-04

**Authors:** Jinzhong Jing, Ying He, Yan Liu, Jiayong Tang, Longqiong Wang, Gang Jia, Guangmang Liu, Xiaoling Chen, Gang Tian, Jingyi Cai, Lianqiang Che, Bo Kang, Hua Zhao

**Affiliations:** 1 Key Laboratory for Animal Disease-Resistance Nutrition of Ministry of Education, of China Ministry of Agriculture and Rural Affairs, of Sichuan Province, Animal Nutrition Institute, Sichuan Agricultural University, Chengdu, 611130 Sichuan China; 2 College of Animal Science and Technology, Sichuan Agricultural University, Chengdu, 611130 Sichuan China

**Keywords:** Dietary oxidative stress, Endoplasmic reticulum stress, Growth retardation, Mitochondrial dysfunction, Selenoproteins, Skeletal muscle

## Abstract

**Background:**

The skeletal muscle of pigs is vulnerable to oxidative damage, resulting in growth retardation. Selenoproteins are important components of antioxidant systems for animals, which are generally regulated by dietary selenium (Se) level. Here, we developed the dietary oxidative stress (DOS)-inducing pig model to investigate the protective effects of selenoproteins on DOS-induced skeletal muscle growth retardation.

**Results:**

Dietary oxidative stress caused porcine skeletal muscle oxidative damage and growth retardation, which is accompanied by mitochondrial dysfunction, endoplasmic reticulum (ER) stress, and protein and lipid metabolism disorders. Supplementation with Se (0.3, 0.6 or 0.9 mg Se/kg) in form of hydroxy selenomethionine (OH-SeMet) linearly increased muscular Se deposition and exhibited protective effects via regulating the expression of selenotranscriptome and key selenoproteins, which was mainly reflected in lower ROS levels and higher antioxidant capacity in skeletal muscle, and the mitigation of mitochondrial dysfunction and ER stress. What's more, selenoproteins inhibited DOS induced protein and lipid degradation and improved protein and lipid biosynthesis via regulating AKT/mTOR/S6K1 and AMPK/SREBP-1 signalling pathways in skeletal muscle. However, several parameters such as the activity of GSH-Px and T-SOD, the protein abundance of JNK2, CLPP, SELENOS and SELENOF did not show dose-dependent changes. Notably, several key selenoproteins such as MSRB1, SELENOW, SELENOM, SELENON and SELENOS play the unique roles during this protection.

**Conclusions:**

Increased expression of selenoproteins by dietary OH-SeMet could synergistically alleviate mitochondrial dysfunction and ER stress, recover protein and lipid biosynthesis, thus alleviate skeletal muscle growth retardation. Our study provides preventive measure for OS-dependent skeletal muscle retardation in livestock husbandry.

**Supplementary Information:**

The online version contains supplementary material available at 10.1186/s40104-023-00877-6.

## Background

Skeletal muscle is the largest organ of pigs, which plays a crucial role in the body shape maintenance and body mobility. Skeletal muscle is also the main product that pigs provide for humans. Normal growth of skeletal muscle is accompanied by the deposition of large amounts of proteins and small amounts of lipids. Mammals are susceptible to oxidative stress (OS) after birth, resulting in skeletal muscle growth retardation, which is usually associated with imbalance of protein and lipid metabolism [1]. Current evidence show that OS induces mitochondrial dysfunction and produces excessive reactive oxygen species (ROS), leading to oxidative damage to skeletal muscle, which is accompanied by decreased protein synthesis and increased protein degradation [2]. Mitochondrial dysfunction impairs the respiratory process of adenosine triphosphate (ATP) synthesis [3], the low levels of ATP cannot provide sufficient energy for protein biosynthesis. Besides, excessive ROS inhibits protein synthesis though regulating the mammalian target of rapamycin (mTOR) pathway [4]. Our previous study found that OS promotes lipolysis [5]. Excessive mitochondrial ROS promotes adenosine monophosphate activated protein kinase (AMPK) phosphorylation and inhibits the expression of sterol regulatory element-binding protein 1 (SREBP-1), thus suppress the fatty acid biosynthesis [6]. Hence, mitochondrial dysfunction may be a major factor that mediates the OS-induced skeletal muscle growth retardation.

The endoplasmic reticulum (ER) provides support for protein and lipid biosynthesis and transportation [7]. Under OS, excessive ROS causes ER stress [8], thus impairing protein and lipid biosynthesis [5, 9]. The ER unfolded protein response (ER-UPR) appears to be the representative biomarker of ER stress [10]. Accumulating evidences posit that three ER-localized transmembrane sensors including protein kinase-like ER kinase (PERK), inositol-requiring protein 1 (IRE1), and activating transcription factor 6 (ATF6) mediate the ER-UPR [11–13]. These sensors promote the occurrence of UPR by activating downstream signalling molecules such as eukaryotic translation initiation factor 2 alpha (eIF2α), activating transcription factor 4 (ATF4), C/EBP homologous protein (CHOP), X-box-binding protein 1 (XBP-1), etc. ER-UPR contributes to eliminate excess unfolded or misfolded peptide chains under stress, thus alleviating the ER stress [14]. Thus, it is reasonable to conjecture that mitigation of mitochondrial dysfunction and ER stress may be an effective strategy to alleviate OS-induced skeletal muscle damage and growth retardation.

Dietary supplementation of antioxidants is an effective way to prevent oxidative damage. As a unique and essential trace element for humans and animals, selenium (Se) is the key component of the body's antioxidant system, which can scavenge excess ROS and improve antioxidant capacity [15, 16]. Se exerts its biological functions in mammals predominantly mediated by selenoproteins [17]. Until now, 25 selenoproteins have been found in mammals, among them, 2 of them (GPX4, TXNRD2) are considered as the mitochondria selenoproteins, and 7 of them (DIO2, SELENOF, SELENOK, SELENOM, SELENON, SELENOS and SELENOT) are located in ER [18]. These selenoproteins are characterized by their ability to regulate mitochondrial and ER homeostasis. As an antioxidant enzyme, GPX4 could eliminate intracellular lipid peroxide [19]. SELENON interacts with ryanodine receptors to form the main component of calcium channels, which are involved in the regulation of ER calcium balance and redox reactions [20, 21]. SELENOS removes the misfolded peptide chains and maintains ER homeostasis [22]. Most of the previous studies are focused on the function of a single selenoprotein. Our current studies on the selenotranscriptome reveal that the biological functions of selenoprotein are coordinated by multiple selenoproteins rather than a single [5, 23, 24].

Generally, the expression of selenoproteins in organs of animals is effectively regulated by dietary Se status [25], and Se concentration in muscle is closely corrected with dietary Se levels in form of organic Se [26, 27]. In present study, the new organic Se molecule (hydroxy selenomethionine, OH-SeMet) was used to regulate the expression of selenoproteins. Oxidized diet (formulated with the maize stored over 4 years and oxidized oils) as stress source to determine: 1) the impact of OS on mitochondrial function, ER homeostasis, and protein and lipid metabolism in skeletal muscle of pigs; 2) the potential mechanism linked to the protective effects of Se and selenoproteins.

## Materials and methods

### Animal, diet and experimental design

The physical properties of the pigs and diets were reported in our previous study [5]. Briefly, 40 crossbreed castrated boars (Duroc × Landrace × Yorkshire) with average body weight of 25.0 ± 3.0 kg were randomly allotted into five dietary treatment groups (*n* = 8), then, these pigs were fed on basal diet (using normal corn and normal oil, without additional Se supplementation, formulated in accordance with NRC 2012, details in Additional file 1: Table S1) for 7 d to adjust the physiological state and balance the body Se level (based on the biological half-life of Se in piglets [28]). After 7 d, the animals were fed on basal diet (control group, CON) or oxidized diet (the normal corn and oil in basal diet were replaced by aged corn stored over 4 years and oxidized oil) supplied with 0.0 (dietary oxidative stress group, DOS), 0.3 (DOS + 0.3 Se), 0.6 (DOS + 0.6 Se) and 0.9 mg Se/kg (DOS + 0.9 Se) in the form of OH-SeMet for 16 weeks. OH-SeMet (hydroxy selenomethionine, Selisso® Adisseo France S.A.S., Paris, France) is kindly provided by Dr. Kevin Liu and Mr. Allen Gao. The determined dietary Se concentration was shown in Additional file 2: Fig. S1. The pigs were penned in fattening circle house with free access to diet and water, and the house temperature was maintained at 25 ± 2 °C and 20 ± 2 °C for 25−50 kg and > 50 kg stages, respectively. The preparation method of oxidized oils was reported in the previous study [29]. The oxidation characteristics of the diets were shown in Additional file 1: Table S2.

### Growth performance

The pigs were weighed every four weeks, after the trial, the average daily gain (ADG) was calculated as follows: ADG = body gain (kg)/test days.

### Feedstuff and blood sample collection

Diets were formulated for four times during the animal experiment. After stored at room temperature (20−30 °C) for two weeks, each batch of diet samples (200 g) from different treatment group were collected and kept at −20 °C for lab analysis. At the beginning (0 d) and the end (16 weeks) of the trial, blood samples were collected in sterile vacutainer tubes from the jugular vein and maintained at room temperature for 1 h, centrifuged (3,000 × *g*) for 10 min, and the serum samples were pipetted into sterile centrifuge tubes, then stored at −20 °C for lab analysis.

### Carcass analysis and *longissimus dorsi* sample collection

At the end of the experiment, a total of thirty pigs (six pigs in each group with a body weight closed to the average body weight) were selected and slaughtered after an overnight fast and sedated by electrical stunning. The carcass weight was estimated as the weight of the hot eviscerated carcass, which was calculated by carcass weight at 45 min after harvest multiplied by 0.98 [30]. The length of the carcass was measured by using a flexible tape on the hanging right half of the carcass at 45 min postmortem, which was the straight distance from the midpoint of the pubic symphysis to the midpoint of the first cervical vertebra. The cross-sectional area of *longissimus dorsi* (LD) at the 12^th^ rib was confirmed: loin-eye area (cm^2^) = loin muscle height (cm) × loin muscle width (cm) × 0.7. Tissue samples of LD were dissected between 12^th^ and 13^th^ ribs and snap-frozen in liquid nitrogen, and stored at − 80 °C.

### Oxidation characteristics of the diet

After diet samples from different batches were mixed, the acid value (AV), peroxide value (POV), iodine value (IV) and saponification value (SV) in diets of each treatment group were determined by using chemical analysis according to the standards (GB 5009.227–2016, GB 5009.229–2016, GB/T 5532–2008 and GB/T 5534–2008) [31–34].

### Selenium concentration in diet, serum and *longissimus dorsi*

Selenium concentration in diets, serum and LD were measured by using the hybrid generation-atomic fluorescence spectrometer (AFS-230E, Beijing Haiguang instrument, China). After diet samples from different batches were mixed, the diets Se concentration of each treatment group were measured based on the standard (GB/T 13883–2008) [35]. Selenium concentration in serum and LD were measured according to the standard (GB 5009.93–2010) [36]. Samples pretreatment have been described previously [26].

### Antioxidant and enzyme analyses

The total glutathione peroxidase (GSH-Px), total antioxidant capability (T-AOC), total superoxide dismutase (T-SOD) and malondialdehyde (MDA) in LD were measured by using the corresponding assay kits (no. A005, A015-1, A001-1–1, A003-1, Nanjin﻿g Jiancheng Bioengineering Institute, China). E3 ubiquitin protein ligase (UBE3) and adipose triglyceride lipase (ATGL) in LD were measured by using the commercial enzyme-linked immunosorbent assay (ELISA) kit (no. MM-7800801, MM-220901, Meimian, Jiangsu, China). The concentration of proteins in each sample was determined with the bicinchoninic acid (BCA) method by using a commercial assay kit (no. A045-3, Nanjing Jiancheng Bioengineering Institute). The optical density values were measured with an ultraviolet–visible spectrophotometer (Model 680, Bio-Rad, Hercules, CA, USA).

### Measurement of ATP and ROS levels in *longissimus dorsi*

The ATP levels in LD samples were determined by using a commercial assay kit (no. A095-1–1, Nanjing Jiancheng Bioengineering Institute, China) according to the manufacturer’s instructions. The concentration of ROS in LD samples was measured using an ELISA kit (no. MM-121201, Meimian, Jiangsu, China) according to the manufacturer’s instructions.

### Q-PCR analyses of mRNA abundance

The total RNA in each LD sample was extracted by using RNAiso Plus (no. 9109, Takara, Dalian, China) and cDNA was synthesized with the PrimeScript RT reagent kit (no. RR047A, Takara, Dalian, China). The qPCR was performed in a final volume of 10 μL using SYBR Premix Ex TaqTM II kit (no. RR820A, Takara, Dalian, China) on the QuantStudio 6 Flex system (Applied Biosystems, CA, USA). The relative mRNA expression was normalized to the expression of *β-Actin* and calculated by using the 2^−ΔΔCt^ method as we used previous [37]. The primers for 22 selenogenes, 8 ER stress biomarkers, 4 ubiquitin-related enzymes, 2 lipid synthesis-related genes and *β-Actin* were designed using Primer Express 3.0 (Applied Biosystems, CA, USA), and listed in Additional file 1: Table S3.

### Western blot analyses

Western blotting process was performed as described previously [23]. In brief, each LD sample was homogenized in ice-cold RIPA buffer with PMSF protease inhibitor buffer (Beyotime, Shanghai, China) and centrifuged (12,000 × *g*) for 30 min at 4 °C. Then, the supernatant of each sample was collected and the total protein concentration was measured using the BCA kit (no. A045-3, Nanjing Jiancheng Bioengineering Institute, China). Protein samples (20 μg) were separated by using 8%−12% SDS-PAGE gels and transferred onto polyvinylidene difluoride membranes (Bio-Rad, CA, USA). The above membranes were blocked with 5% defatted milk for 2 h and washed three times with TBST (prepared with Tris–HCl buffer and isotonic salt solution and contains 1% Tween 20) and incubated overnight at 4 °C with primary antibodies (detailed in Additional file 1: Table S4). Then, these membranes were incubated with corresponding secondary antibodies (anti-rabbit or anti-mouse IgG, 1:5,000, Proteintech Group, IL, USA). The bands were visualized by using an enhanced chemiluminescence system (Bio-Rad, CA, USA), and the densitometric of Western blot bands were analyzed using the Image Lab™ software system (Bio-Rad, Hercules, CA, USA).

### Statistical analysis

This study was followed the complete random design (CRD) and applied the one-way structure treatment design. All data are expressed as means ± standard errors. Statistical analyses were performed with the SPSS 27.0 (SPSS Inc., Chicago, USA), values were analyzed using one-way ANOVA followed by Tukey’s multiple range tests, and ANOVA *P*-values of less than 0.05 were considered statistically significant. Principal component analysis of selenotranscriptome in LD and correlation analysis between the key selenoproteins and other indicators were accomplished by SPSS 27.0 (SPSS, Inc., Chicago, USA). All results were plotted using GraphPad Prism Version 8 software (Graphpad software, LLC, San Diego, USA).

## Results

### Selenium concentration in serum and *longissimus dorsi*

**Fig. 1:**
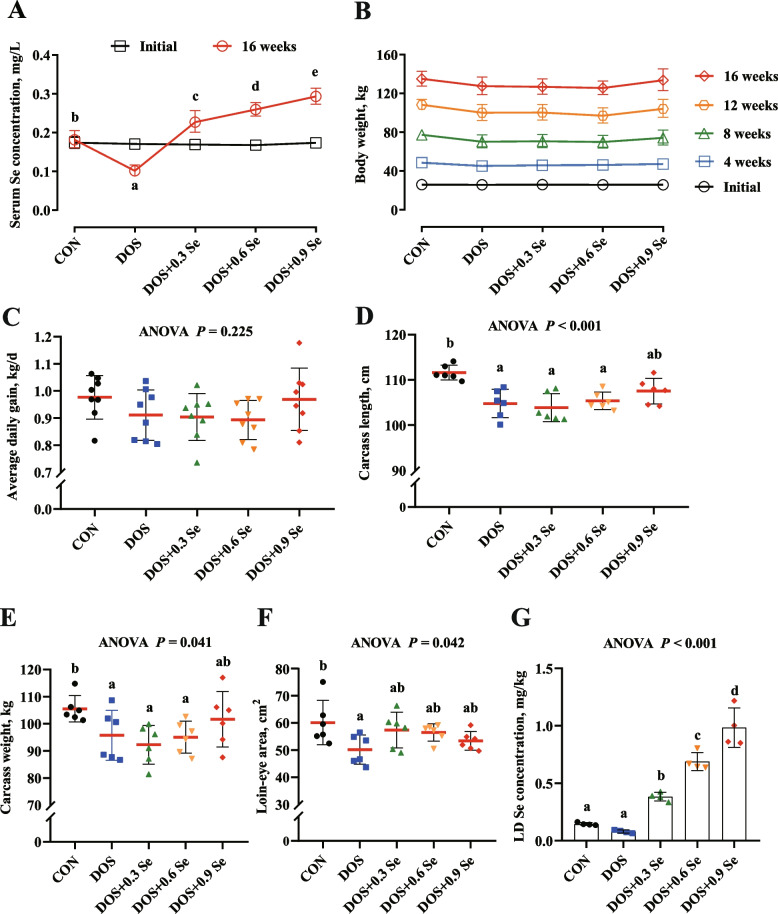
Effects of DOS and OH-SeMet supplementation on serum Se level, growth performance, carcass traits, LD cross-sectional area and LD Se concentration of pigs. **A** Serum Se concentration; **B** Body weight; **C** Average daily gain; **D** Carcass length; **E** Carcass weight; **F** Loin-eye area; **G** LD Se concentration. Results were expressed as mean ± SD (*n* = 8 for performance, 6 for carcass traits and 4 for Se concentration), different letters indicate significant differences (*P* < 0.05)

At the beginning of the trial, pigs in each treatment were in the similar serum Se status (0 d) (Fig. 1A). After 16 weeks of treatment, dietary oxidative stress (DOS) decreased (*P* < 0.05) the Se level in serum (Fig. 1A) and reduced Se deposition in LD (Fig. 1G). Dietary supplementation of OH-SeMet linearly increased (*P* < 0.05) Se concentrations in serum and LD (Fig. 1A, G).

### Growth performance, carcass traits and* longissimus dorsi* cross-sectional area

To determine the effect of DOS and OH-SeMet supplementation on growth performance of pigs, we recorded the body weight at different stages and measured the carcass traits and the cross-sectional area of LD. As shown in Fig. 1C and B, although there were no statistically significant differences, DOS numerically reduced the ADG and body weight at different stages. Dietary supplementation of 0.9 mg Se/kg OH-SeMet alleviated the negative effects of DOS on growth performance. Slaughter results showed that DOS lowered (*P* < 0.05) the carcass length, carcass weight and loin-eye area of pigs (Fig. 1D, E and F). While, Se supplementation especially 0.9 mg Se/kg improved the carcass traits and the loin-eye area of pigs faced with DOS.

### Antioxidant capacity of *longissimus dorsi*

**Fig. 2:**
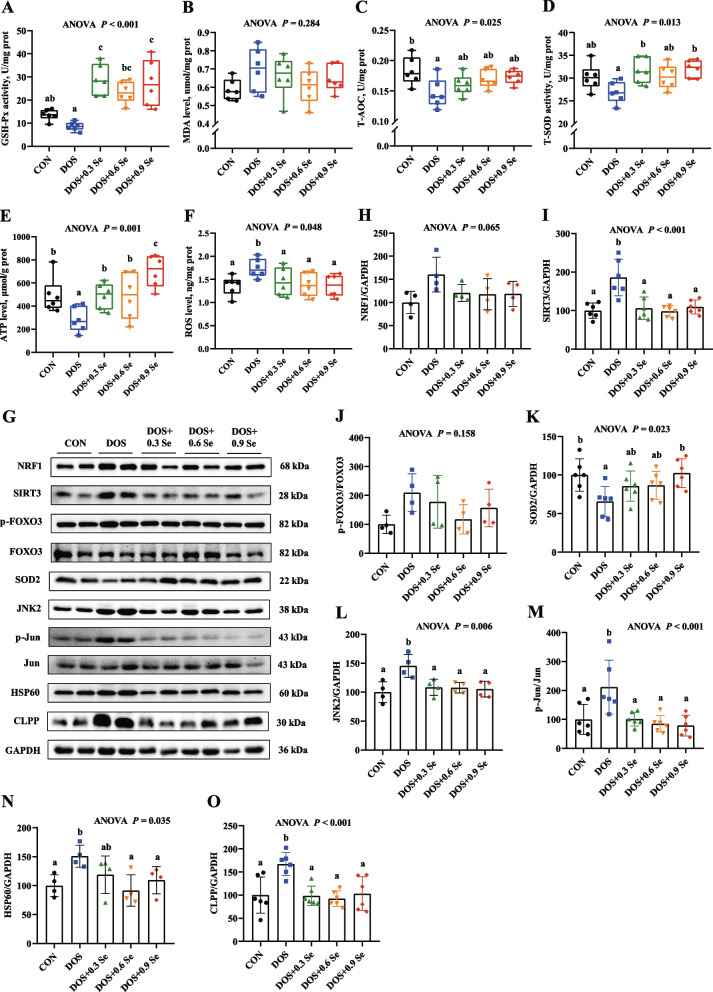
Effects of DOS and OH-SeMet supplementation on antioxidant variables, ATP levels, ROS levels and mitochondrial stress biomarkers in LD. **A** GSH-Px activity; **B** MDA levels; **C** T-AOC activity; **D** T-SOD activity; **E** ATP levels; **F** ROS levels; **G** Protein expression of mitochondrial stress biomarkers; **H** Relative protein abundance of NRF1; **I** Relative protein abundance of SIRT3; **J** Relative protein abundance of p-FOXO3; **K** Relative protein abundance of SOD2; **L** Relative protein abundance of JNK2; **M** Relative protein abundance of p-Jun; **N** Relative protein abundance of HSP60; **O** Relative protein abundance of CLPP. Results were expressed as mean ± SD (*n* = 6 or 4), different letters indicate significant differences (*P* < 0.05)

We measured the antioxidant capacity of LD to evaluate whether the prolonged DOS causes oxidative damage to skeletal muscle and the protective effects of Se. Results showed that DOS decreased the antioxidant capacity in LD, these changes were accompanied by the lower activities of GSH-Px, T-AOC and T-SOD (Fig. 2A, C and D). Dietary Se supplementation exhibited protective effects, as reflected by the increased or recovered (*P* < 0.05) activities of GSH-Px, T-AOC and T-SOD in LD. While, these changes were not dose-dependent with the OH-SeMet levels, except T-AOC.

### ATP and ROS levels in *longissimus dorsi*

To evaluate whether DOS induces mitochondria damage and the protective effects of Se, we determined the ATP and ROS levels in LD. Compared with the CON group, pigs faced with DOS showed a lower ATP level and a higher ROS level in LD (Fig. 2E and F). Dietary Se supplementation relieved the negative effect of DOS on the levels of ATP and ROS, which linearly increased (*P* < 0.05) the ATP levels and decreased (*P* < 0.05) the ROS levels.

### Expression of mitochondrial unfolded protein response biomarkers in *longissimus dorsi*

The biomarkers of mitochondrial unfolded protein response (MT-UPR) in LD were determined (Fig. 2G). Relative to the CON group, DOS decreased (*P* < 0.05) the protein level of SOD2 (Fig. 2K), while increased (*P* < 0.05) the protein levels of NAD-dependent protein deacetylase sirtuin 3 (SIRT3), c-Jun N-terminal kinase 2 (JNK2), phosphorylated transcription factor AP1 (p-Jun), heat shock protein 60 (HSP60) and caseinolytic mitochondrial matrix peptidase (CLPP) (Fig. 2I, L, M, N and O). Besides, DOS tended to increase (0.05 < *P* < 0.1) the level of nuclear respiratory factor 1 (NRF1) (Fig. 2H). Dietary Se supplementation exhibited protective effects, which were reflected in the increased protein level of SOD2 and the decreased (*P* < 0.05) protein abundance of SIRT3, CLPP, JNK2, p-Jun, HSP60 and NRF1. However, these changes were not dose-dependent with the OH-SeMet levels. Besides, DOS and Se supplementation showed no effects (*P* > 0.05) on the protein abundances of p-FOXO3.

### Expression of ER stress biomarkers in *longissimus dorsi*

**Fig. 3:**
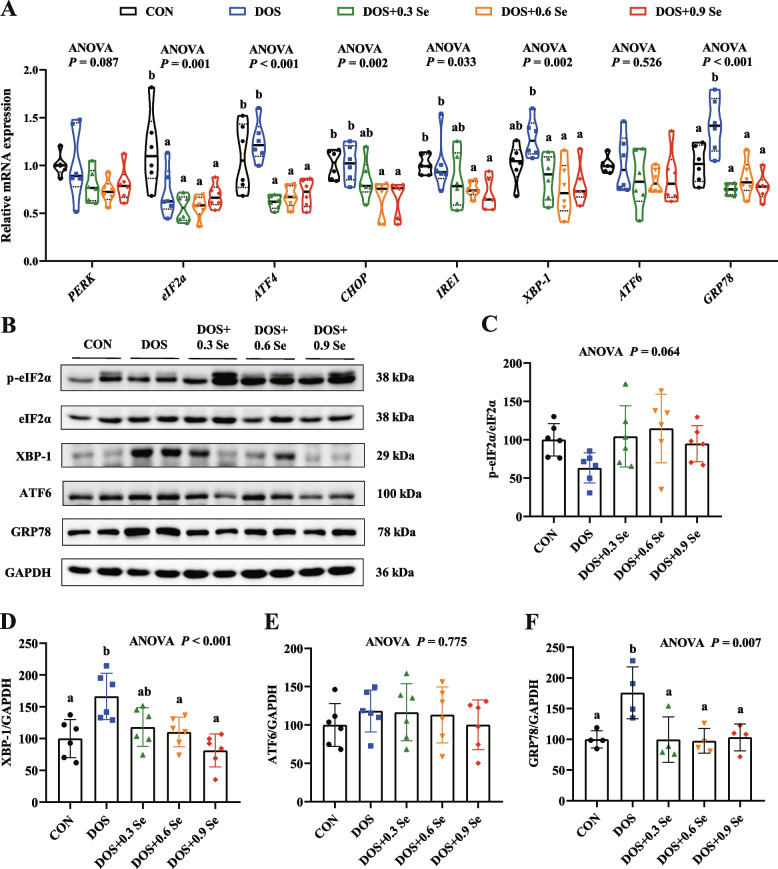
Effects of DOS and OH-SeMet supplementation on the expression of ER stress biomarkers in LD. **A** The mRNA expression of *PERK*, *eIF2α*, *ATF4*, *CHOP*, *IRE1*, *XBP-1*, *ATF6* and *GRP78*; **B** Protein expression of ER stress biomarkers; **C** Relative protein abundance of p-eIF2α; **D** Relative protein abundance of XBP-1; **E** Relative protein abundance of ATF6; **F** Relative protein abundance of GRP78. Results were expressed as mean ± SD (*n* = 6 or 4), different letters indicate significant differences (*P* < 0.05)

We further determined the ER stress biomarkers in LD (Fig. 3). Firstly, we determined the mRNA expression of 8 ER-UPR markers (Fig. 3A). Compared with the CON group, DOS down-regulated (*P* < 0.05) the mRNA expression of *eIF2α*, up-regulated (*P* < 0.05) *GRP78* level, and tended to increase (0.05 < *P* < 0.1) *XBP-1* level. Dietary Se supplementation decreased (*P* < 0.05) the mRNA abundance of *ATF4*, *CHOP*, *IRE1*, *XBP-1* and *GRP78*. DOS and Se supplementation exhibited limited effects (*P* > 0.05) on the mRNA expression of *PERK* and *ATF6*.

We further examined the protein expression of four ER stress biomarkers (Fig. 3B). Results showed that DOS increased (*P* < 0.05) the protein abundance of XBP-1 and GRP78 (Fig. 3D and F), and tended to decrease (0.05 < *P* < 0.1) p-eIF2α (Fig. 3C). Dietary Se supplementation recovered (*P* < 0.05) the protein levels of XBP-1 and GRP78, while tended to increase the protein abundance of p-eIF2α. While, these changes were not dose-dependent with the OH-SeMet levels except XBP-1. Besides, DOS and Se supplementation showed no effects on the protein expression of ATF6 (Fig. 3E).

### Protein metabolism indicators in *longissimus dorsi*

**Fig. 4:**
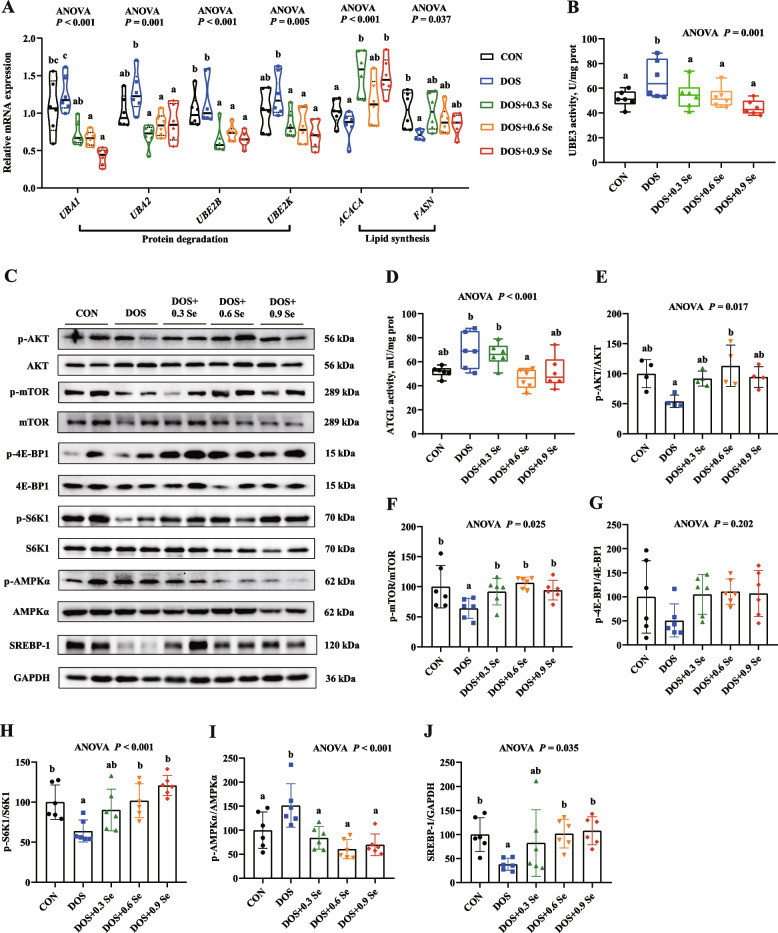
Effects of DOS and OH-SeMet supplementation on the protein metabolism and lipid metabolism in LD. **A** Relative mRNA expression of *UBA1*, *UBA2*, *UBE2B*, *UBE2K*, *ACACA* and *FASN*; **B** Activity of UBE3; **C** Protein expression of AKT-mTOR and AMPK-SREBP-1 signalling pathway; **D** Activity of ATGL; **E** Relative protein abundance of p-AKT; **F** Relative protein abundance of p-mTOR; **G** Relative protein abundance of p-4E-BP1; **H** Relative protein abundance of p-S6K1; **I** Relative protein abundance of p-AMPKα; **J** Relative protein abundance of SREBP-1. Results were expressed as mean ± SD (*n* = 6 or 4), different letters indicate significant differences (*P* < 0.05)

The protein metabolism indicators in LD were determined (Fig. 4). Relative to the CON group, long-term DOS promoted protein degradation in skeletal muscle, which is mainly reflected in the up-regulation of *UBA1*, *UBA2* and *UBE2K* mRNA levels (Fig. 4A), and the increased activity of UBE3 (Fig. 4B). Besides, DOS decreased the relative protein abundance of p-AKT, p-mTOR and p-S6K1 (Fig. 4E, F, H), thus suppressed the AKT-mTOR-S6K signalling pathway and led to protein biosynthesis inhibition in LD. Dietary Se supplementation reversed the negative impact of DOS. Pigs received additional dietary Se showed the lower *UBA1*, *UBA2* and *UBE2K* mRNA profiles and UBE3 activity (*P* < 0.05). Se supplementation also increased (*P* < 0.05) the protein expression of p-AKT, p-mTOR and p-S6K1 (Fig. 4E, F and H). While, several parameters such as the *UBE2B* levels, p-AKT and p-mTOR abundances did not showed the dose-dependent relationship with OH-SeMet levels.

### Lipid metabolism indicators in *longissimus dorsi*

We further investigated the lipid metabolism indicators in LD of pigs (Fig. 4). DOS affected the AMPK-SREBP-1 signalling pathway, which is mainly reflected in the up-regulation (*P* < 0.05) of p-AMPKα protein level (Fig. 4I) and down-regulation (*P* < 0.05) of SREBP-1 protein level (Fig. 4J) and mRNA expression of *FASN* (Fig. 4A), thus suppressed the lipid biosynthesis in LD. Besides, DOS increased (0.05 < *P* < 0.1) the activity of ATGL (Fig. 4D). Dietary Se supplementation showed positive impacts on the lipid metabolism indicators in LD under DOS. OH-SeMet supplementation recovered (*P* < 0.05) the protein levels of p-AMPKα, increased (*P* < 0.05) the protein expression of SREBP-1, up-regulated (*P* < 0.05) the mRNA levels of *ACACA* and *FASN*. Besides, 0.6 and 0.9 mg Se/kg OH-SeMet decreased (*P* < 0.05) the activity of ATGL. While, several indicators such as *ACACA* levels, ATGL activity and p-AMPKα abundance did not showed the dose-dependent relationship with OH-SeMet levels.

### Expression of selenotranscriptome in *longissimus dorsi*

**Fig. 5:**
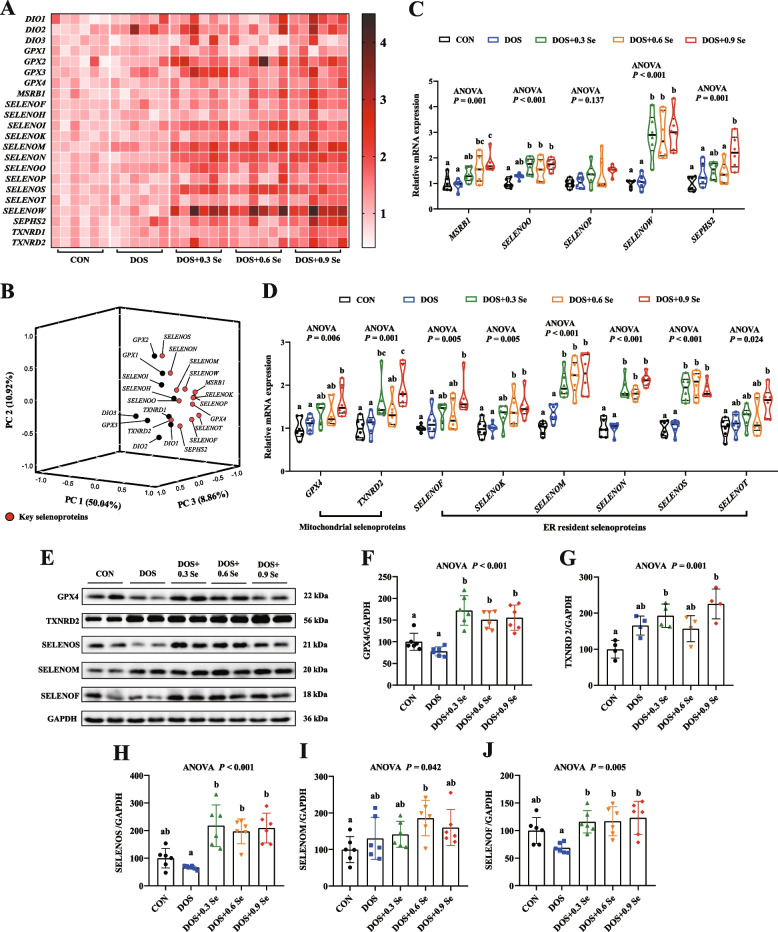
Effects of DOS and OH-SeMet supplementation on the expression of selenotranscriptome and 5 key selenoproteins in LD. **A** Heatmap of mRNA abundance of selenotranscriptome; **B** Principal component analysis of the selenotranscriptome; **C** and **D** Relative mRNA expression of 13 key selenogenes; **E** Protein expression of 5 key selenoproteins; **F** Relative protein abundance of GPX4; **G** Relative protein abundance of Txnrd2; **H** Relative protein abundance of SelS; **I** Relative protein abundance of SelM; **J** Relative protein abundance of SelF. Results were expressed as mean ± SD (*n* = 6 or 4), different letters indicate significant differences (*P* < 0.05)

Se exerts its most-known biological functions mainly through selenoproteins. Here, the selenotranscriptome in LD of pigs were determined. *GPX6*, *SELENOV* and *TXNRD3* in skeletal muscle were in poor quality or low expression and were not reported herein. DOS alone showed limited impact on the expression of these selenogenes, except up-regulated (*P* < 0.05) the expression of *DIO2* and *GPX4* (Fig. 5A, detailed profiles are shown in Additional file 3: Fig. S2). Dietary Se supplementation especially 0.9 mg Se/kg increased (*P* < 0.05) the abundance of 15 selenogenes (*DIO1*, *GPX2*, *GPX3*, *MSRB1*, *SELENOI*, *SELENOK*, *SELENOM*, *SELENON*, *SELENOO*, *SELENOS*, *SELENOT*, *SELENOW*, *SEPHS2*, *TXNRD1* and *TXNRD2*) in LD of pigs faced with DOS. Besides, DOS and Se supplementation exhibited limited impacts (*P* > 0.05) on the expression of *DIO3*, *GPX1*, *SELENOH* and *SELENOP*.

### Expression of key selenoproteins in *longissimus dorsi*

We further performed a principal component analysis through the mathematical method of dimensionality reduction to distinguish the key selenoproteins affected by DOS and OH-SeMet. Three comprehensive variables were chosen to reflect the original variable information (Fig. 5B). Results showed that 13 selenogenes were observed at relatively distant positions in three-dimensional space. Thus, these 13 selenogenes (*GPX4*, *MSRB1*, *SELENOF*, *SELENOK*, *SELENOM*, *SELENON*, *SELENOO*, *SELENOP*, *SELENOS*, *SELENOT*, *SELENOW*, *SEPHS2*, *TXNRD2*) were the key genes affected by DOS and OH-SeMet. Compared with the DOS group, these key genes showed the higher mRNA levels in Se supplementation groups, except *SELENOP* (Fig. 5C and D). Among these key genes, 2 of them (GPX4, TXNRD2) are considered as the mitochondria selenoproteins, 6 of them (SELENOF, SELENOK, SELENOM, SELENON, SELENOS, SELENOT) are located in the ER.

Thus, we further determined the protein abundance of GPX4, TXNRD2, SELENOF, SELENOM and SELENOS in LD (Fig. 5E). DOS tended to increase (0.05 < *P* < 0.1) the relative protein levels of TXNRD2 and SELENOM (Fig. 5G and I), while tended to decrease (0.05 < *P* < 0.1) the protein levels of SELENOF and SELENOS (Fig. 5J and H). Compared with the DOS group, Se supplementation increased (*P* < 0.05) the relative protein abundance of GPX4, SELENOF and SELENOS, and tended to increase (0.05 < *P* < 0.1) the levels of TXNRD2 and SELENOM. Interestingly, the protein abundance of these 5 selenoproteins did not showed the dose-dependent relationship with OH-SeMet levels.

### Correlation analysis

**Fig. 6:**
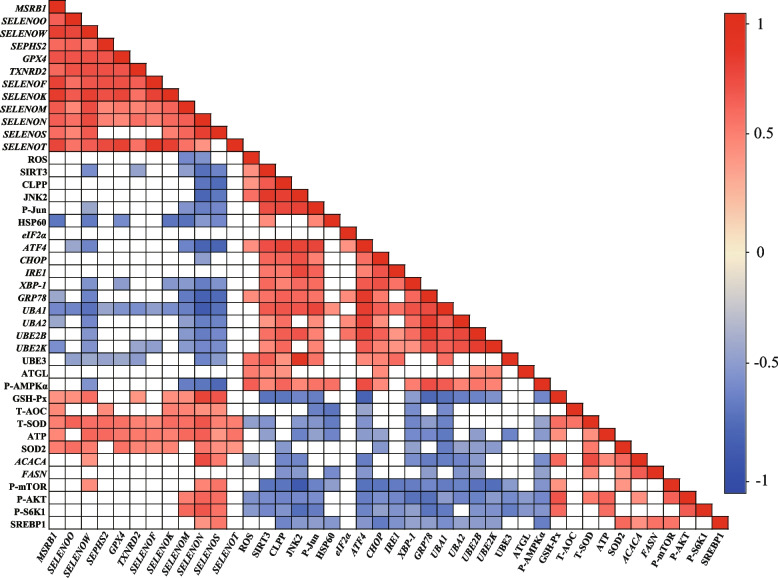
Correlation analysis between the key selenogenes and other measures. Pearson correlation coefficients were provided in Additional file 1: Table S5. The color of red represents a significant positive correlation (*P* < 0.05), blue represents a significant negative correlation (*P* < 0.05)

Correlation analysis was further conducted to estimate the potential relationship between the key selenogenes and other measures that were changed significantly under DOS and OH-SeMet supplementation (details are provided in Additional file 1: Table S5). As shown in Fig. 6, significant positive correlations were found among most of the 12 key selenogenes. Three genes (*SELENOM*, *SELENON*, *SELENOS*) exhibited the primary negative correlations with the mitochondrial stress biomarkers (ROS, SIRT3, CLPP, JNK2, p-Jun and HSP60), while exhibited the primary positive correlations with the protein synthesis indicators (p-AKT, p-mTOR, p-S6K1). Four genes (*SELENOW*, *SELENOM*, *SELENON*, *SELENOS*) were negatively correlated with ER stress biomarkers (*ATF4*, *XBP-1*, *GRP78*). Five genes (*MSRB1*, *SELENOW*, *SELENOM*, *SELENON*, *SELENOS*) exhibited the primary negative correlations with protein degradation indicators (*UBA1*, *UBA2*, *UBE2B*, *UBE2K*, UBE3). Almost all the key selenogenes were positively correlated with the antioxidant variables (GSH-Px, T-AOC, T-SOD, SOD2) and ATP. Besides, *SELENON* and *SELENOS* were positively correlated with the lipid synthesis indicators (SREBP-1 and *ACACA*).

## Discussion

Dietary oxidative stress comprises rancid oil, moldy food, aged grain, etc., which is a common oxidative stressor in animal husbandry, these diets is rich in lipid peroxidation products after heating and/or long-term storage [38, 39], thus cause oxidative damage to animals. Current evidences suggest that lipid peroxidation product intake induces OS and promotes the disease progression and impairs the growth of skeletal muscle [1, 40]. In present study, a long-term DOS was administered to the growing-finishing pigs to assess the negative effect of OS on skeletal muscle growth. Pigs faced with DOS exhibited the numerically lower body weight in each period and the lower ADG, carcass length and carcass weight. Skeletal muscle accounts for more than 50% of a pig's body weight [41], it is reasonable to expect that DOS affects skeletal muscle growth. LD is the key component of porcine skeletal muscle, and its cross-sectional area can used to effectively evaluate the skeletal muscle growth status of pigs [42]. Similarly, DOS decreased the LD cross-sectional area. These results suggest that long-term DOS causes skeletal muscle growth retardation in pigs. Selenium is considered to be an excellent antioxidant, and Se concentration in muscle is closely corrected with dietary Se levels in form of organic Se [26, 27]. In this study OH-SeMet supplementation increased the serum and LD Se concentration in a dose dependent pattern. Likewise, dietary OH-SeMet supplementation especially 0.9 mg Se/kg increased the body weight, carcass length, carcass weight and LD cross-sectional area of pigs under DOS. Therefore, organic Se supplementation in form of OH-SeMet alleviates skeletal muscle growth retardation of pigs suffered with DOS.

Oxidative damage is the major factor that causes skeletal muscle growth retardation. The main cause of oxidative damage is the excessive ROS produced by dysfunctional mitochondria, which exceeds the carrying capacity of the antioxidant system [43]. Mitochondrial dysfunction impairs the oxidative phosphorylation process and affects the ATP biosynthesis [44, 45]. Here, we further evaluated the oxidative damage status in skeletal muscle of pigs faced with DOS and Se. In present study, DOS decreased the antioxidant capacity of LD, which is mainly reflected in the lower activities of total GSH-Px, T-AOC and T-SOD. Besides, DOS decreased the ATP level and increased the ROS level in LD, which suggest that DOS causes mitochondrial dysfunction and induces oxidative damage in skeletal muscle. Interestingly, dietary Se supplementation decreased the ROS levels and increased ATP levels, which is alongside with the improvement of antioxidant capacity in LD.

To explore whether the potential protective effect of Se is associated with alleviation of mitochondrial dysfunction, we measured the expression of MT-UPR biomarkers in LD. MT-UPR is a conserved signalling pathway in mammals and has gained extensive attention as an important regulatory pathway for mitochondrial communication [46]. Under stress conditions, the MT-UPR senses insufficient protein-handling capacity in mitochondria and aims to restore mitochondrial homeostasis by expanding the folding capacity and enhancing protein degradation [47]. There are three distinct branches involved in the MT-UPR signalling pathway in mammals [46, 48]. One pathway is that excessive ROS phosphorylates AKT and regulates the expression of mitochondrial regulator NRF1, thus initiating MT-UPR. Mitochondrial dysfunction also activates SIRT3, which induces deacetylation of FOXO3 into the nucleus and enhances the transcription of the ROS detoxification gene *SOD2*. Besides, accumulation of unfolded proteins in the mitochondrial matrix can activate transcription factor Jun by JNK2, phosphorylated Jun leading to the induction of CHOP and CCAAT/enhancer-binding protein-β (CEBPβ), thus initiating MT-UPR. Molecular chaperones and proteases in the mitochondrial matrix, such as HSP60, CLPP, constitute major targets of MT-UPR signalling. Enhancing their expression could effectively scavenge unfolded proteins from the mitochondrial matrix, thus maintaining mitochondrial function [49, 50]. In present study, DOS decreased the protein level of SOD2, increased SIRT3, JNK2, p-Jun, HSP60 and CLPP. Dietary Se supplementation recovered the protein abundance of SOD2 and decreased the protein levels of SIRT3, JNK2, p-Jun, HSP60 and CLPP, thus alleviates mitochondrial dysfunction, and this protective process is alongside with regulation of SIRT3/FOXO3/SOD2 and JNK2/Jun/CHOP signalling pathways.

Under OS, excessive ROS produced by dysfunctional mitochondria generally causes ER stress and ER dysfunction, thus affects the biosynthesis of protein and lipid [8, 9]. ER-UPR is the representative behavior of the ER stress [51]. Under ER stress, abundance of unfolded proteins accumulated in the ER facilitate the activation of ER-UPR through three ER transmembrane sensors: PERK, IRE1 and ATF6 [11–13]. These sensors contribute to the restoration of ER homeostasis by regulating multiple downstream signalling molecules such as eIF2α, ATF4, CHOP and XBP-1, and molecular chaperone such as GRP78 is the major target of ER-UPR signalling in the ER lumen [52]. ER stress generally increases the expression of these biomarkers. In this study, DOS increased the abundance of XBP-1 and GRP78 at mRNA and protein levels, and decreased the mRNA expression of *eIF2α* in LD, indicating that DOS causes ER stress and promotes the occurrence of ER-UPR. Pigs received three levels of OH-SeMet showed the lower *ATF4*, *CHOP*, *IRE1*, *XBP-1* and *GRP78* mRNA levels, and the lower XBP-1 and GRP78 protein levels in LD. These results ulteriorly reveal that OH-SeMet contributes to improve the ER homeostasis in skeletal muscle under DOS through regulating the ER-UPR, thus improves protein and lipid metabolism homeostasis.

The maintenance of mitochondrial and ER homeostasis provides guarantee for protein and lipid synthesis, and OH-SeMet supplementation effectively alleviates mitochondrial dysfunction and ER stress in skeletal muscle under OS. These results prompted us to further investigate the effects of Se on protein and lipid metabolism in skeletal muscle under DOS. For protein metabolism, DOS increased the mRNA expression of *UBA1*, *UBA2* and *UBE2K*, and increased the activity of UBE3 in LD. The ubiquitin–proteasome pathway plays a unique role in the degradation of skeletal muscle proteins [53]. In this process, ubiquitin molecules are activated by ubiquitin-activating enzyme (UBE1) and transported to ubiquitin-conjugating enzyme (UBE2), UBE2 and UBE3 cooperate to bind ubiquitin molecules to proteins, thus degrading proteins [54]. UBA1 and UBA2 are common subtypes of UBE1, UBE2B and UBE2K are common subtypes of UBE2. Our results suggest that DOS promotes protein degradation in porcine skeletal muscle. mTOR pathway has gained attention as a key regulator of protein biosynthesis [55]. Phosphorylated mTOR inhibits 4E-BP1 and activates S6K to promote the translation of ribosomal S6 [56, 57]. And mTOR pathway is generally regulated by the upstream signal AKT. Evidence shows that excessive ROS inhibits protein synthesis though regulating the 4E-BP1 and S6K [4]. In present study, DOS suppressed the protein abundance of p-AKT, p-mTOR and p-S6K1, suggesting DOS inhibits protein synthesis in skeletal muscle. Interestingly, dietary OH-SeMet supplementation suppressed protein degradation and promoted protein biosynthesis in skeletal muscle, which were accompanied by the down-regulation of *UBA1*, *UBA2*, *UBE2B* and *UBE2K* mRNA levels, and the lower activity of UBE3, while the increase of p-AKT, p-mTOR and p-S6K1 protein levels. For lipid metabolism, we found that DOS decreased the mRNA expression of *FASN* and the protein abundance of SREBP-1, and increased the activity of ATGL and the protein expression of p-AMPK. FAS encoded by *FASN* is the key enzyme that regulates fatty acid synthesis, which promotes the elongation of fatty acid chains [58]. ACC encoded by *ACACA* is the rate-limiting enzyme in the process of fatty acid de novo synthesis, similar to FAS, ACC is regulated by nuclear transcription factor SREBP-1 [59, 60]. The activation of SREBP-1 is usually regulated by AMPK. ATGL facilitate the specifically hydrolyzation of the first ester bond of triglyceride, which is considered to be the rate-limiting enzyme in the lipolysis [61]. Our present study confirms that DOS suppresses the lipid biosynthesis and promotes lipolysis in LD of pigs. Dietary Se supplementation exhibited protective effects, which are reflected in the up-regulation of *ACACA*, *FASN* mRNA levels and SREBP-1 protein levels, the lower ATGL activity and recovered protein expression of p-AMPK. Based on these results, OH-SeMet supplementation improves protein and lipid metabolism homeostasis in skeletal muscle of pigs under DOS, and this process is medicated with the regulating of AKT/mTOR/S6K1 and AMPK/SREBP-1 pathway.

Our previous studies of the selenotranscriptome in different animal models suggest that Se performs its biological function through the synergy of multiple selenoproteins [5, 23, 26, 62]. Hence, we explored the mRNA expression of selenotranscriptome in the skeletal muscle and identified key selenogenes using principal component analysis. Thirteen among 22 selenogenes were identified as the key genes response to DOS and OH-SeMet supplementation. Among these selenoproteins, GPX4 and TXNRD2 are considered as the mitochondria selenoproteins, SELENOF, SELENOK, SELENOM, SELENON, SELENOS and SELENOT are located in ER, while the other 4 selenoproteins (MSRB1, SELENOP, SELENOW, SEPHS2) are in a free state [18]. At present, the location and function of SELENOO are remain unclear, study suggests that SELENOO located in the mitochondria and is associated with redox control [63]. MSRB1 was found to control the assembly and disassembly of actin in mammals, suggesting it may be involved in skeletal muscle growth regulation [64]. What's more, MSRB1 participates in the regulation of redox homeostasis and protect proteins from oxidative damage [65]. SELENOP is mainly responsible for the transport of Se, which can transport Se from plasma to various target organs, thus controlling the expression of all selenoproteins alongside with SEPHS2 [66]. About the mitochondrial selenoproteins, GPX4 could eliminate intracellular lipid peroxide, TXNRD2 protects against disulfide damage and regulates redox homeostasis, lack of GPX4 and TXNRD2 is lethal in mice [18, 67]. About the ER resident selenoproteins, most of them are involved in the regulation of ER homeostasis. SELENOF participates in the protein quality control by mediating disulfide bond formation, which can clear excess unfolded or misfolded proteins under ER stress conditions [68, 69]. SELENON interacts with ryanodine receptors to form the main component of calcium channels, which is involved in the regulation of ER calcium balance and redox reactions [20, 21]. Besides, previous study found that SELENON affects skeletal muscle development, and knockout of the SELENON causes the destruction of the muscle structure in zebrafish embryonic [70]. SELENOS is the central component of retro-translocation channel in ER-associated protein degradation, which can remove misfolded peptide chains and maintains ER homeostasis [22, 71]. SELENOK, SELENOM and SELENOT control the ER homeostasis though scavenging excessive ROS and suppressing apoptosis [72–75]. In the present study, DOS alone exhibited limited impact on the mRNA expression of these selenoproteins, while tended to increase the protein abundance of TXNRD2 and SELENOM, and tended to decrease the protein levels of SELENOS and SELENOF. Dietary OH-SeMet supplementation up-regulated the expression of these key selenoproteins at mRNA or protein levels, except *SELENOP*. Based on the above results, it is clear that OH-SeMet alleviates skeletal muscle growth retardation induced by DOS though regulating the expression of 12 key selenogenes.

We further performed correlation analysis to demonstrate the synergistic effect of selenoproteins and the relationship between 12 key selenogenes and these assayed indicators. The significant positive correlations were found among most of the 12 genes, and almost all the genes were positively correlated with the antioxidant variables (GSH-Px, T-AOC, T-SOD, SOD2) and ATP levels, which validates our hypothesis that Se performs its biological function through the synergy of multiple selenoproteins. Interestingly, the results of correlation analysis showed that a subset selenogenes showed significant positive correlation in the process of alleviating mitochondrial dysfunction and ER stress, such as *MSRB1*, *SELENOW*, *SELENOM*, *SELENON* and *SELENOS*, and lipid and protein metabolism-related indicators analysis showed the similar results. Based on these results, it is reasonable to believe that dietary OH-SeMet supplementation improves lipid and protein metabolism in skeletal muscle of pigs under DOS mainly through regulating a few key selenoproteins (MSRB1, SELENOW, SELENOM, SELENON and SELENOS), while the other selenoproteins play an auxiliary role in this process.

## Conclusion

**Fig. 7:**
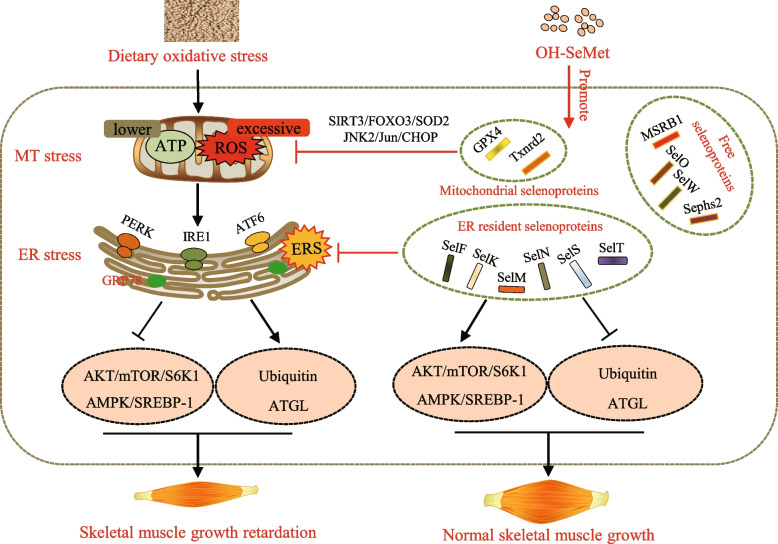
Schematic diagram of selenoproteins alleviating skeletal muscle growth retardation of pigs induced by DOS. Several key selenoproteins synergistically scavenge excessive ROS, release mitochondrial and ER stress, and restore the inhibition of protein and lipid biosynthesis caused by dietary oxidative stress, thus alleviate skeletal muscle growth retardation

In present study, we reveal that the DOS-induced skeletal muscle retardation in pigs is associated with mitochondrial dysfunction and ER stress, which suppresses the protein and lipid biosynthesis. Dietary supplementation of organic Se in form of OH-SeMet linearly increases the deposition of Se in skeletal muscle, enabling skeletal muscle quickly mobilize Se reserves to synthesize selenoproteins under stress. Multiple in vivo evidences suggest that increased expression of selenoproteins alleviate mitochondrial dysfunction and ER stress, recover protein and lipid biosynthesis, thus alleviate skeletal muscle retardation. Several key selenoproteins exhibit synergistic effects in this process. Our results contribute to better understanding on the synergistic mechanism of key selenoproteins against OS-induced skeletal muscle growth retardation in mammals (Fig. 7), which provide preventive measure for OS-dependent skeletal muscle retardation in livestock husbandry.

## Supplementary Information

**Additional file 1: Table S1.** Composition and nutrient levels of the basal diet. **Table S2.** The oxidation Characteristics of the diets. **Table S3.** Primers used for the Q-PCR. **Table S4.** Primary antibodies for the Western blot analyses. **Table S5.** The correlation analysis between the 12 key selenogenes and other measures.

**Additional file 2: Fig. S1.** Diet Se concentration.

**Additional file 3: Fig. S2.** The mRNA expression of the 22 selenogenes.

## Data Availability

All data generated or analyzed during this study are included in this published article and its supplementary information files.

## Abbreviations

ADG: Average daily gain
AMPK: Adenosine monophosphate activated protein kinase
ATF4: Activating transcription factor 4
ATF6: Activating transcription factor 6
ATGL: Adipose triglyceride lipase
ATP: Adenosine triphosphate
AV: Acid value
CHOP: C/EBP homologous protein
CLPP: Caseinolytic mitochondrial matrix peptidase
DOS: Dietary oxidative stress
eIF2α: Eukaryotic translation initiation factor 2 alpha
ER: Endoplasmic reticulum
ER-UPR: Endoplasmic reticulum unfolded protein response
FOXO3: Forkhead box O3
GSH-Px: Glutathione peroxidase
HSP60: Heat shock protein 60
IRE1: Inositol-requiring protein 1
IV: Iodine value
JNK2: C-Jun N-terminal kinase 2
Jun: Transcription factor AP1
LD: *Longissimus dorsi*
MDA: Malondialdehyde
mTOR: Mammalian target of rapamycin
MT-UPR: Mitochondrial unfolded protein response
NRF1: Nuclear respiratory factor 1
OH-SeMet: Hydroxy selenomethionine
p-AMPKα: Phosphorylated adenosine monophosphate activated protein kinase alpha
p-eIF2α: Phosphorylated eukaryotic translation initiation factor 2 alpha
PERK: Protein kinase-like endoplasmic reticulum kinase
p-FOXO3: Phosphorylated forkhead box O3
p-Jun: Phosphorylated transcription factor AP1
POV: Peroxide value
ROS: Reactive oxygen species
SIRT3: NAD-dependent protein deacetylase sirtuin 3
SREBP-1: Sterol regulatory element-binding protein 1
SV: Saponification value
T-AOC: Total antioxidant capability
T-SOD: Total superoxide dismutase
UBE3: E3 ubiquitin protein ligase
XBP-1: X-box-binding protein 1
