# Supplementary material for: Selenoproteins synergistically protect porcine skeletal muscle from oxidative damage via relieving mitochondrial dysfunction and endoplasmic reticulum stress
Source: J Anim Sci Biotechnol. 2023 Jun 4;14:79. doi: 10.1186/s40104-023-00877-6 (PMC10239589; doi:10.1186/s40104-023-00877-6)
Supplement: Supplementary file 1 — Additional file 1: Table S1. Composition and nutrient levels of the basal diet. Table S2. The oxidation Characteristics of the diets. Table S3. Primers used for the Q-PCR. Table S4. Primary antibodies for the Western blot analyses. Table S5. The correlation analysis between the 12 key selenogenes and other measures. [file 40104_2023_877_MOESM1_ESM.docx]

**Additional Table Information**

**Table S1** Composition and nutrient levels of the basal diet (air-dry basis), %

| **Ingredients** | **Phase** | |
| --- | --- | --- |
|  | **25–50 kg** | **> 50 kg** |
| Corn | 73.610 | 70.525 |
| Soybean meal | 17.900 | 6.500 |
| Fish meal | 2.700 | - |
| Rapeseed dregs | - | 6.500 |
| DDGS | - | 8.000 |
| Wheat bran | - | 1.000 |
| Corn oil | 2.000 | 3.000 |
| Fish oil | 1.000 | 2.000 |
| CaHPO_4_ | 0.670 | 0.550 |
| Limestone | 0.930 | 0.900 |
| NaCl | 0.300 | 0.200 |
| *L*-lysine HCl | 0.440 | 0.470 |
| *DL*-Methionine | 0.100 | 0.030 |
| *L*-Threonine | 0.120 | 0.090 |
| *L*-Tryptophan | 0.030 | 0.035 |
| Premix^1^ | 0.200 |  |
| Premix^2^ |  | 0.200 |
| Total | 100 | 100 |
| Nutrient composition | | |
| DE, Mcal/kg | 3.40 | 3.48 |
| CP, % | 15.69 | 13.64 |
| Ca, % | 0.66 | 0.53 |
| AP, % | 0.31 | 0.24 |
| SID Lys, % | 0.98 | 0.74 |
| SID Met+cys, % | 0.54 | 0.43 |
| SID Thr, % | 0.59 | 0.47 |
| SID Trp, % | 0.17 | 0.14 |

^1^Premix for 25-50 kg pig provided (per kg): Cu (CuSO_4_·5H_2_O), 4 mg; I (KI), 0.14 mg; Fe (FeSO_4_·7H_2_O), 60 mg; Mn (MnSO_4_·H_2_O), 2 mg; Zn (ZnSO_4_·H_2_O), 60 mg; 50% Choline chloride, 75 mg; Vitamin A, 1,500 IU; Vitamin D_3_, 500 IU; Vitamin E, 4 mg; Vitamin K_3_, 0.5 mg; Vitamin B_1_, 0.5 mg; Vitamin B_2_, 1.25 mg; Vitamin B_6_, 0.6 mg; Vitamin B_12_, 6 μg; *D*-biotin, 25 μg; D-pantothenic acid, 2.5 mg; Folic acid, 0.25 mg; Nicotinamide, 5 mg

^2^Premix for > 50 kg pig provided (per kg): Cu (CuSO_4_·5H_2_O), 7 mg; I (KI), 0.2 mg; Fe (FeSO_4_·7H_2_O), 100 mg; Mn (MnSO_4_·H_2_O), 4 mg; Zn (ZnSO_4_·H_2_O), 50 mg; 50% Choline chloride, 200 mg; Vitamin A, 450 IU; Vitamin D_3_, 150 IU; Vitamin E, 1.2 mg; Vitamin K_3_, 0.15 mg; Vitamin B_1_, 0.15 mg; Vitamin B_2_, 0.375 mg; Vitamin B_6_, 0.18 mg; Vitamin B_12_, 1.8 μg; *D*-biotin, 7.5 μg; *D*-pantothenic acid, 0.75 mg; Folic acid, 75 μg; Nicotinamide, 1.5 mg

**Table S2** The oxidation Characteristics of the diets

| **Item** | **CON** | **DOS** | **DOS + 0.3 Se** | **DOS + 0.6 Se** | **DOS + 0.9 Se** |
| --- | --- | --- | --- | --- | --- |
| Diet for 25-50 kg | | | | | |
| AV mg KOH/100 g | 56.70 | 238.90 | 238.40 | 233.30 | 240.20 |
| POV mmol O_2_/kg | 19.60 | 433.60 | 415.70 | 429.20 | 414.60 |
| IV, g I/100 g | 126.70 | 81.20 | 83.90 | 83.50 | 82.10 |
| SV, mg KOH/g | 182.10 | 237.70 | 240.60 | 248.30 | 242.70 |
| Diet for > 50 kg | | | | | |
| AV, mg KOH/100 g | 68.00 | 252.60 | 253.70 | 264.60 | 257.40 |
| POV, mmol O_2_/kg | 31.80 | 519.20 | 523.60 | 537.80 | 534.20 |
| IV, g I/100 g | 111.70 | 73.40 | 73.60 | 74.70 | 72.80 |
| SV, mg KOH/g | 195.40 | 261.30 | 265.70 | 268.20 | 266.70 |

AV, acid value; POV, peroxide value; IV, iodine value; SV, saponification value

**Table S3** Primers used for the Q-PCR

| **Gene** | **Accession number** | **Primer pairs (5′ to 3′ direction)** |  |
| --- | --- | --- | --- |
| *β-Actin* | AY550069 | F: CCCAAAGCCAACCGTGAGAA |  |
|  |  | R: CCACGTACATGGCTGGGGTG |  |
| *UBA1* | XM_021080357.1 | F: AGCCTCCTCTCAGTATGTCCA |  |
|  |  | R: AGGGGAACAGTTAGAACCCG |  |
| *UBA2* | XM_021097539.1 | F: AACCTCCGGTTCCTTTGGAC |  |
|  |  | R: TCAGACCTAACTGGGGTTCGT |  |
| *UBE2B* | NM_001257356.1 | F: GGCGCACCATCTGAAAACAA |  |
|  |  | R: AACAGTTGGCGGTTTATTTGGA |  |
| *UBE2K* | NM_001078671.1 | F: CAGCGAATCAAGCGGGAGT |  |
|  |  | R: TTGATTTTTGCTCGTCTCCTCG |  |
| *ACACA* | NM_001114269.1 | F: CAAGACCACCAACGCGAAA |  |
|  |  | R: GGCAAATGGGAGGCAATAAGA |  |
| *FASN* | NM_001099930 | F: CAAGCAGGCGAACACGATG |  |
|  |  | R: AAGGGAAGCAGGGTTGATGC |  |
|  |  | R: AGATGTCCGAAGCCATGTCC |  |
| *PERK* | XM_003124925.4 | F: CTGTACCCATTCGGCACTCA |  |
|  |  | R: ATGTCCTGCACCATCGCAAA |  |
| *eIF2α* | XM_001928339.4 | F: AATAGGCGTTTGACCCCACA |  |
|  |  | R: TGTTCTCTCCAGGGTGGTTG |  |
| *ATF4* | NM_001123078.1 | F: ATGCCCTGTCGGGTATAGATGA |  |
|  |  | R: ATCCAACGTGGCCAAAAGC |  |
| *CHOP* | NM_001144845.1 | F: GGAAATGAGGAGGAGTCAAAAACC |  |
|  |  | R: CTCAGTCAGCCAAGCCAGAGA |  |
| *IRE1* | XM_005668695.3 | F: CTGAGCGAAGACTGCAAGGA |  |
|  |  | R: GAGTATGTTGGCCTGACGCT |  |
| *XBP-1* | NM_001142836.1 | F: CAGACTGCCAGAGACCGAAAGA |  |
|  |  | R: TCTTCCAAATCTACCACTTGTTGCT |  |
| *ATF6* | XM_021089516.1 | F: GGGAGTGAGCTGCAGGTGTATT |  |
|  |  | R: TCTGCGGATGGCTTCAAAGA |  |
| *GRP78* | XM_001927795.7 | F: TGGGAAAGAAGGTTACTCATGCA |  |
|  |  | R: CTGGCGTTGGGCATCATT |  |
| **Selenoprotein-encoding genes** | | |  |
|  |  |  |  |
| *DIO1* | NM_001001627 | F: CATGGCCAAGAACCCTCACT |  |
|  |  | R: CCAGAAATACTGGGCACTGAAGA |  |
| *DIO2* | NM_001001626 | F: CGCTGCATCTGGAAGAGCTT |  |
|  |  | R: TGGAATTGGGTGCATCTTCA |  |
| *DIO3* | NM_001001625 | F: TGAAGTGGAGCTCAACAGTGATG |  |
|  |  | R: TGTCGTCAGACACGCAGATAGG |  |
| *GPX1* | NM_214201 | F: GATGCCACTGCCCTCATGA |  |
|  |  | R: TCGAAGTTCCATGCGATGTC |  |
| *GPX2* | NM_001115136 | F: AGAATGTGGCCTCGCTCTGA |  |
|  |  | R: GGCATTGCAGCTCGTTGAG |  |
| *GPX3* | NM_001115155 | F: CCGGTTCCTGTTTTCCAAATT |  |
|  |  | R: TGCACTGCAGGAAGAGTTTGAA |  |
| *GPX4* | NM_214407 | F: TGAGGCAAGACGGAGGTAAACT |  |
|  |  | R: TCCGTAAACCACACTCAGCATATC |  |
| *MSRB1* | NM_001097460 | F: ATCCCTAAAGGCCAAGAATCATC |  |
|  |  | R: GGCCACCAAGCAGTGTTCA |  |
| *SELENOF* | NM_001085443 | F: ACAGCCCTGCCAAGCAGAT |  |
|  |  | R: AACAGGGAGGCTGGGTAACAC |  |
| *SELENOH* | NM_001184948 | F: TGGTGGAGGAGCTGAAGAAGTAC |  |
|  |  | R: CGTCATAAATGCTCCAACATCAC |  |
| *SELENOI* | NM_001244662 | F: GATGGTGTGGATGGAAAGCAA |  |
|  |  | R: GCCATGGTCAAAGAGTTCTCCTA |  |
| *SELENOK* | NM_001044553 | F: CAGGAAACCCCCCTAGAAGAA |  |
|  |  | R: CTCATCCACCGGCCATTG |  |
| *SELENOM* | NM_001161648 | F: CAGCTGAATCGCCTCAAAGAG |  |
|  |  | R: GAGATGTTTCATGACCAGGTTGTG |  |
| *SELENON* | XM_021095600 | F: ACCTGGTCCCTGGTGAAAGAG |  |
|  |  | R: AGGCCAGCCAGCTTCTTGT |  |
| *SELENOO* | NM_001201431 | F: CTTCCGACCCCAGATGGAT |  |
|  |  | R: GGTTCGACTGTGCCAGCAT |  |
| *SELENOP* | NM_001134823 | F: AACCAGAAGCGCCAGACACT |  |
|  |  | R: TGCTGGCATATCTCAGTTCTCAGA |  |
| *SELENOS* | NM_001164113 | F: GAGGCAGAGGCACCTGGAT |  |
|  |  | R: CTGCTAAAGCCTCCTGTCGTTT |  |
| *SELENOT* | NM_001163408 | F: GGCTTAATAATCGTTGGCAAAGA |  |
|  |  | R: TGGCCCCATTGCCAGATA |  |
| *SELENOW* | NM_213977 | F: CACCCCTGTCTCCCTGCAT |  |
|  |  | R: GAGCAGGATCACCCCAAACA |  |
| *SEPHS2* | NM_001093735 | F: TGGCTTGATGCACACGTTTAA |  |
|  |  | R: TGCGAGTGTCCCAGAATGC |  |
| *TXNRD1* | NM_214154 | F: GATTTAACAAGCGGGTCATGGT |  |
|  |  | R: CAACCTACATTCACACACGTTCCT |  |
| *TXNRD2* | NM_001168702 | F: TCTTGAAAGGCGGAAAAGAGAT |  |
|  |  | R: TCGGTCGCCCTCCAGTAG |  |

**Table S4** Primary antibodies for the western blot analyses

| **Antibody** | **Company** | **Address** | **Commodity code** | **Dilution ratio** |
| --- | --- | --- | --- | --- |
| NRF1 | Cell signaling technology | Massachusetts, USA | 46743 | 1:1,000 |
| p-mTOR | Cell signaling technology | Massachusetts, USA | 5536 | 1:1,000 |
| mTOR | Cell signaling technology | Massachusetts, USA | 2983 | 1:1,000 |
| p-4EBP1 | Cell signaling technology | Massachusetts, USA | 2855 | 1:1,000 |
| 4EBP1 | Cell signaling technology | Massachusetts, USA | 9644 | 1:1,000 |
| p-S6K1 | Cell signaling technology | Massachusetts, USA | 9209 | 1:1,000 |
| S6K1 | Cell signaling technology | Massachusetts, USA | 9202 | 1:1,000 |
| p-AMPKα | Cell signaling technology | Massachusetts, USA | 2535 | 1:1,000 |
| AMPKα | Cell signaling technology | Massachusetts, USA | 5831 | 1:1,000 |
| SIRT3 | Zen BioScience | Chengdu, China | R25724 | 1:1,000 |
| p-FOXO3 | Zen BioScience | Chengdu, China | R24347 | 1:1,000 |
| FOXO3 | Zen BioScience | Chengdu, China | R26154 | 1:1,000 |
| SOD2 | Zen BioScience | Chengdu, China | 206028 | 1:1,000 |
| CLPP | Zen BioScience | Chengdu, China | R23947 | 1:1,000 |
| JNK2 | Zen BioScience | Chengdu, China | 160527 | 1:1,000 |
| p-Jun | Zen BioScience | Chengdu, China | R22955 | 1:1,000 |
| Jun | Zen BioScience | Chengdu, China | R23335 | 1:1,000 |
| XBP-1 | Zen BioScience | Chengdu, China | R27438 | 1:1,000 |
| p-eIF2α | Zen BioScience | Chengdu, China | 310073 | 1:500 |
| eIF2α | Zen BioScience | Chengdu, China | 340347 | 1:500 |
| p-AKT | Zen BioScience | Chengdu, China | R22961 | 1:1,000 |
| AKT | Zen BioScience | Chengdu, China | R23411 | 1:1,000 |
| SREBP-1 | Zen BioScience | Chengdu, China | 347061 | 1:500 |
| SELENOM | Zen BioScience | Chengdu, China | 821208 | 1:500 |
| GPX4 | Zen BioScience | Chengdu, China | 513309 | 1:2,000 |
| SELENOF | Zen BioScience | Chengdu, China | 385690 | 1:1,000 |
| ATF6 | Proteintech Group | Illinois, USA | 66563-1-Ig | 1:5,000 |
| GRP78 | Proteintech Group | Illinois, USA | 66574-1-Ig | 1:5,000 |
| HSP60 | Proteintech Group | Illinois, USA | 66041-1-Ig | 1:5,000 |
| TXNRD2 | Proteintech Group | Illinois, USA | 16360-1-AP | 1:1,000 |
| SELENOS | Proteintech Group | Illinois, USA | 15591-1-AP | 1:1,000 |
| GAPDH | Proteintech Group | Illinois, USA | 60004-1-Ig | 1:10,000 |

**Table S5** The correlation analysis between the 12 key selenogenes and other measures

| **Correlations** | | | | | | | | | | | | | | | | | | | | | | | | | | | | | | | | | | | | | | | | | | | |
| --- | --- | --- | --- | --- | --- | --- | --- | --- | --- | --- | --- | --- | --- | --- | --- | --- | --- | --- | --- | --- | --- | --- | --- | --- | --- | --- | --- | --- | --- | --- | --- | --- | --- | --- | --- | --- | --- | --- | --- | --- | --- | --- | --- |
|  |  | MSRB1 | SELENOO | SELENOW | SEPHS2 | GPX4 | TXNRD2 | SELENOF | SELENOK | SELENOM | SELENON | SELENOS | SELENOT | ROS | SIRT3 | CLPP | JNK2 | p-Jun | HSP60 | eIF2a | ATF4 | CHOP | IRE1 | XBP1 | GRP78 | UBA1 | UBA2 | UBE2B | UBE2K | UBE3 | ATGL | p-AMPK | GSH-Px | T-AOC | T-SOD | ATP | SOD2 | ACACA | FASN | PmTOR | p-AKT | p-S6K1 | SREBP1 |
| MSRB1 | Pearson Correlation | 1 | .654^**^ | .825^**^ | .609^**^ | .711^**^ | .615^**^ | .815^**^ | .867^**^ | .680^**^ | .658^**^ | .598^**^ | .788^**^ | -0.275 | -0.363 | -0.273 | -0.213 | -0.351 | -.659^**^ | 0.061 | -0.359 | -0.103 | -0.151 | -0.295 | -0.399 | -.567^**^ | -0.397 | -0.325 | -.537^**^ | -0.323 | -0.307 | -0.373 | .425^*^ | .466^*^ | .513^*^ | .475^*^ | .519^**^ | 0.257 | 0.110 | 0.382 | 0.291 | 0.373 | 0.302 |
|  | Sig. (2-tailed) |  | 0.001 | 0.000 | 0.002 | 0.000 | 0.001 | 0.000 | 0.000 | 0.000 | 0.000 | 0.002 | 0.000 | 0.194 | 0.081 | 0.197 | 0.427 | 0.092 | 0.005 | 0.778 | 0.085 | 0.633 | 0.482 | 0.161 | 0.053 | 0.004 | 0.055 | 0.121 | 0.007 | 0.124 | 0.144 | 0.073 | 0.039 | 0.022 | 0.010 | 0.019 | 0.009 | 0.225 | 0.610 | 0.065 | 0.273 | 0.072 | 0.152 |
| SELENOO | Pearson Correlation | .654^**^ | 1 | .777^**^ | .636^**^ | .708^**^ | .718^**^ | .585^**^ | .671^**^ | .474^*^ | .582^**^ | .494^*^ | .562^**^ | -0.160 | -0.352 | -0.167 | -0.170 | -0.274 | -0.356 | 0.007 | -.406^*^ | -0.154 | -0.205 | -0.321 | -0.355 | -.582^**^ | -0.373 | -0.275 | -0.248 | -.441^*^ | -0.104 | -0.236 | .444^*^ | 0.233 | .633^**^ | 0.367 | .589^**^ | 0.402 | 0.173 | 0.210 | 0.117 | 0.121 | 0.295 |
|  | Sig. (2-tailed) | 0.001 |  | 0.000 | 0.001 | 0.000 | 0.000 | 0.003 | 0.000 | 0.019 | 0.003 | 0.014 | 0.004 | 0.455 | 0.091 | 0.436 | 0.529 | 0.195 | 0.176 | 0.973 | 0.049 | 0.473 | 0.336 | 0.127 | 0.089 | 0.003 | 0.073 | 0.193 | 0.242 | 0.031 | 0.629 | 0.266 | 0.030 | 0.273 | 0.001 | 0.077 | 0.002 | 0.051 | 0.419 | 0.326 | 0.667 | 0.574 | 0.162 |
| SELENOW | Pearson Correlation | .825^**^ | .777^**^ | 1 | .566^**^ | .723^**^ | .736^**^ | .695^**^ | .827^**^ | .747^**^ | .757^**^ | .680^**^ | .665^**^ | -0.291 | -.542^**^ | -0.330 | -0.301 | -0.403 | -.633^**^ | -0.289 | -.582^**^ | -0.228 | -0.151 | -.512^*^ | -.588^**^ | -.678^**^ | -.568^**^ | -.499^*^ | -.516^**^ | -0.400 | -0.220 | -.512^*^ | .584^**^ | 0.403 | .583^**^ | .619^**^ | .526^**^ | .416^*^ | 0.225 | .450^*^ | 0.376 | 0.344 | 0.257 |
|  | Sig. (2-tailed) | 0.000 | 0.000 |  | 0.004 | 0.000 | 0.000 | 0.000 | 0.000 | 0.000 | 0.000 | 0.000 | 0.000 | 0.167 | 0.006 | 0.116 | 0.257 | 0.051 | 0.008 | 0.170 | 0.003 | 0.285 | 0.482 | 0.011 | 0.002 | 0.000 | 0.004 | 0.013 | 0.010 | 0.053 | 0.301 | 0.011 | 0.003 | 0.051 | 0.003 | 0.001 | 0.008 | 0.043 | 0.290 | 0.027 | 0.152 | 0.100 | 0.225 |
| SEPHS2 | Pearson Correlation | .609^**^ | .636^**^ | .566^**^ | 1 | .703^**^ | .725^**^ | .727^**^ | .722^**^ | .473^*^ | .503^*^ | 0.214 | .763^**^ | -0.123 | -0.145 | 0.155 | -0.186 | -0.156 | -0.420 | 0.218 | -0.058 | -0.052 | -0.166 | -0.385 | -0.167 | -.417^*^ | -0.105 | 0.005 | -0.035 | -.425^*^ | -0.011 | -0.069 | 0.223 | .457^*^ | .597^**^ | .604^**^ | 0.342 | 0.335 | -0.082 | -0.034 | 0.286 | 0.318 | 0.103 |
|  | Sig. (2-tailed) | 0.002 | 0.001 | 0.004 |  | 0.000 | 0.000 | 0.000 | 0.000 | 0.020 | 0.012 | 0.316 | 0.000 | 0.566 | 0.498 | 0.469 | 0.491 | 0.468 | 0.105 | 0.306 | 0.787 | 0.810 | 0.437 | 0.063 | 0.434 | 0.043 | 0.626 | 0.980 | 0.871 | 0.038 | 0.958 | 0.747 | 0.295 | 0.025 | 0.002 | 0.002 | 0.102 | 0.110 | 0.703 | 0.876 | 0.283 | 0.130 | 0.632 |
| GPX4 | Pearson Correlation | .711^**^ | .708^**^ | .723^**^ | .703^**^ | 1 | .704^**^ | .725^**^ | .796^**^ | .539^**^ | .474^*^ | 0.323 | .821^**^ | -0.227 | -0.376 | -0.070 | -0.172 | -0.312 | -.553^*^ | 0.087 | -0.264 | -0.189 | -0.255 | -.470^*^ | -0.231 | -.510^*^ | -0.206 | -0.288 | -0.326 | -0.399 | -0.137 | -0.293 | 0.329 | 0.290 | .576^**^ | .549^**^ | .574^**^ | 0.263 | 0.188 | 0.348 | 0.278 | 0.146 | 0.318 |
|  | Sig. (2-tailed) | 0.000 | 0.000 | 0.000 | 0.000 |  | 0.000 | 0.000 | 0.000 | 0.007 | 0.019 | 0.123 | 0.000 | 0.285 | 0.070 | 0.747 | 0.525 | 0.138 | 0.026 | 0.687 | 0.213 | 0.377 | 0.229 | 0.021 | 0.278 | 0.011 | 0.335 | 0.172 | 0.121 | 0.054 | 0.524 | 0.164 | 0.116 | 0.169 | 0.003 | 0.005 | 0.003 | 0.214 | 0.378 | 0.096 | 0.297 | 0.497 | 0.130 |
| TXNRD2 | Pearson Correlation | .615^**^ | .718^**^ | .736^**^ | .725^**^ | .704^**^ | 1 | .651^**^ | .577^**^ | .493^*^ | .582^**^ | 0.317 | .571^**^ | -0.168 | -.433^*^ | -0.042 | -0.262 | -0.238 | -0.293 | -0.007 | -0.341 | -0.225 | -0.267 | -0.384 | -0.361 | -.541^**^ | -0.368 | -0.311 | -.410^*^ | -.468^*^ | -0.121 | -0.222 | .417^*^ | 0.317 | .508^*^ | .537^**^ | .489^*^ | 0.349 | 0.036 | 0.246 | 0.120 | 0.299 | 0.177 |
|  | Sig. (2-tailed) | 0.001 | 0.000 | 0.000 | 0.000 | 0.000 |  | 0.001 | 0.003 | 0.014 | 0.003 | 0.131 | 0.004 | 0.433 | 0.034 | 0.844 | 0.328 | 0.263 | 0.270 | 0.973 | 0.102 | 0.291 | 0.207 | 0.064 | 0.083 | 0.006 | 0.077 | 0.139 | 0.046 | 0.021 | 0.574 | 0.297 | 0.043 | 0.131 | 0.011 | 0.007 | 0.015 | 0.095 | 0.866 | 0.246 | 0.658 | 0.157 | 0.407 |
| SELENOF | Pearson Correlation | .815^**^ | .585^**^ | .695^**^ | .727^**^ | .725^**^ | .651^**^ | 1 | .814^**^ | .543^**^ | .502^*^ | 0.257 | .902^**^ | 0.003 | -0.204 | -0.043 | -0.009 | -0.214 | -0.274 | -0.035 | -0.195 | -0.033 | -0.116 | -0.333 | -0.275 | -.451^*^ | -0.388 | -0.319 | -.453^*^ | -0.157 | -0.135 | 0.001 | 0.310 | 0.373 | .457^*^ | .524^**^ | .449^*^ | 0.223 | 0.010 | 0.224 | 0.121 | 0.273 | 0.068 |
|  | Sig. (2-tailed) | 0.000 | 0.003 | 0.000 | 0.000 | 0.000 | 0.001 |  | 0.000 | 0.006 | 0.012 | 0.225 | 0.000 | 0.991 | 0.340 | 0.841 | 0.974 | 0.316 | 0.305 | 0.870 | 0.361 | 0.879 | 0.588 | 0.112 | 0.193 | 0.027 | 0.061 | 0.128 | 0.026 | 0.465 | 0.529 | 0.995 | 0.141 | 0.073 | 0.025 | 0.009 | 0.028 | 0.294 | 0.964 | 0.292 | 0.656 | 0.197 | 0.751 |
| SELENOK | Pearson Correlation | .867^**^ | .671^**^ | .827^**^ | .722^**^ | .796^**^ | .577^**^ | .814^**^ | 1 | .629^**^ | .610^**^ | .516^**^ | .827^**^ | -0.141 | -0.346 | -0.149 | -0.144 | -0.348 | -.664^**^ | 0.018 | -0.315 | -0.109 | -0.146 | -.491^*^ | -0.373 | -.540^**^ | -0.393 | -0.277 | -0.349 | -0.258 | -0.141 | -0.328 | .439^*^ | .509^*^ | .597^**^ | .509^*^ | .487^*^ | 0.301 | 0.174 | 0.349 | 0.405 | 0.262 | 0.286 |
|  | Sig. (2-tailed) | 0.000 | 0.000 | 0.000 | 0.000 | 0.000 | 0.003 | 0.000 |  | 0.001 | 0.002 | 0.010 | 0.000 | 0.511 | 0.098 | 0.487 | 0.596 | 0.096 | 0.005 | 0.932 | 0.133 | 0.611 | 0.496 | 0.015 | 0.072 | 0.006 | 0.057 | 0.190 | 0.095 | 0.223 | 0.512 | 0.117 | 0.032 | 0.011 | 0.002 | 0.011 | 0.016 | 0.154 | 0.415 | 0.094 | 0.120 | 0.217 | 0.175 |
| SELENOM | Pearson Correlation | .680^**^ | .474^*^ | .747^**^ | .473^*^ | .539^**^ | .493^*^ | .543^**^ | .629^**^ | 1 | .651^**^ | .619^**^ | .571^**^ | -.564^**^ | -.453^*^ | -0.304 | -0.384 | -.516^**^ | -.683^**^ | -0.253 | -.588^**^ | -0.190 | -0.102 | -.479^*^ | -.632^**^ | -.603^**^ | -.452^*^ | -.483^*^ | -.478^*^ | -0.387 | -0.380 | -.614^**^ | .448^*^ | .499^*^ | .580^**^ | .609^**^ | 0.189 | 0.294 | 0.109 | 0.256 | .513^*^ | .615^**^ | 0.251 |
|  | Sig. (2-tailed) | 0.000 | 0.019 | 0.000 | 0.020 | 0.007 | 0.014 | 0.006 | 0.001 |  | 0.001 | 0.001 | 0.004 | 0.004 | 0.026 | 0.149 | 0.142 | 0.010 | 0.004 | 0.233 | 0.003 | 0.375 | 0.635 | 0.018 | 0.001 | 0.002 | 0.027 | 0.017 | 0.018 | 0.062 | 0.067 | 0.001 | 0.028 | 0.013 | 0.003 | 0.002 | 0.375 | 0.164 | 0.612 | 0.227 | 0.042 | 0.001 | 0.237 |
| SELENON | Pearson Correlation | .658^**^ | .582^**^ | .757^**^ | .503^*^ | .474^*^ | .582^**^ | .502^*^ | .610^**^ | .651^**^ | 1 | .830^**^ | .424^*^ | -.510^*^ | -.694^**^ | -.666^**^ | -.747^**^ | -.605^**^ | -0.487 | -0.240 | -.760^**^ | -.454^*^ | -0.359 | -.615^**^ | -.772^**^ | -.826^**^ | -.612^**^ | -.618^**^ | -.570^**^ | -.587^**^ | -0.336 | -.652^**^ | .805^**^ | .444^*^ | .697^**^ | .649^**^ | .547^**^ | .744^**^ | 0.352 | .493^*^ | .629^**^ | .691^**^ | .423^*^ |
|  | Sig. (2-tailed) | 0.000 | 0.003 | 0.000 | 0.012 | 0.019 | 0.003 | 0.012 | 0.002 | 0.001 |  | 0.000 | 0.039 | 0.011 | 0.000 | 0.000 | 0.001 | 0.002 | 0.056 | 0.260 | 0.000 | 0.026 | 0.085 | 0.001 | 0.000 | 0.000 | 0.001 | 0.001 | 0.004 | 0.003 | 0.109 | 0.001 | 0.000 | 0.030 | 0.000 | 0.001 | 0.006 | 0.000 | 0.092 | 0.014 | 0.009 | 0.000 | 0.039 |
| SELENOS | Pearson Correlation | .598^**^ | .494^*^ | .680^**^ | 0.214 | 0.323 | 0.317 | 0.257 | .516^**^ | .619^**^ | .830^**^ | 1 | 0.219 | -0.387 | -.587^**^ | -.716^**^ | -.644^**^ | -.567^**^ | -.561^*^ | -0.181 | -.756^**^ | -0.384 | -0.376 | -.510^*^ | -.721^**^ | -.687^**^ | -.590^**^ | -.564^**^ | -.543^**^ | -.475^*^ | -0.264 | -.732^**^ | .698^**^ | .444^*^ | .509^*^ | 0.401 | .442^*^ | .529^**^ | 0.324 | .554^**^ | .595^*^ | .570^**^ | .541^**^ |
|  | Sig. (2-tailed) | 0.002 | 0.014 | 0.000 | 0.316 | 0.123 | 0.131 | 0.225 | 0.010 | 0.001 | 0.000 |  | 0.303 | 0.061 | 0.003 | 0.000 | 0.007 | 0.004 | 0.024 | 0.398 | 0.000 | 0.064 | 0.070 | 0.011 | 0.000 | 0.000 | 0.002 | 0.004 | 0.006 | 0.019 | 0.213 | 0.000 | 0.000 | 0.030 | 0.011 | 0.052 | 0.030 | 0.008 | 0.122 | 0.005 | 0.015 | 0.004 | 0.006 |
| SELENOT | Pearson Correlation | .788^**^ | .562^**^ | .665^**^ | .763^**^ | .821^**^ | .571^**^ | .902^**^ | .827^**^ | .571^**^ | .424^*^ | 0.219 | 1 | -0.129 | -0.123 | 0.059 | 0.036 | -0.148 | -0.407 | 0.011 | -0.108 | 0.016 | -0.007 | -0.365 | -0.209 | -0.384 | -0.243 | -0.216 | -0.299 | -0.176 | -0.077 | -0.097 | 0.148 | 0.375 | .515^*^ | .556^**^ | .415^*^ | 0.233 | 0.047 | 0.165 | 0.139 | 0.165 | 0.075 |
|  | Sig. (2-tailed) | 0.000 | 0.004 | 0.000 | 0.000 | 0.000 | 0.004 | 0.000 | 0.000 | 0.004 | 0.039 | 0.303 |  | 0.550 | 0.566 | 0.786 | 0.896 | 0.491 | 0.117 | 0.959 | 0.617 | 0.941 | 0.973 | 0.080 | 0.327 | 0.064 | 0.252 | 0.310 | 0.156 | 0.410 | 0.721 | 0.652 | 0.489 | 0.071 | 0.010 | 0.005 | 0.044 | 0.273 | 0.827 | 0.440 | 0.607 | 0.440 | 0.728 |
| ROS | Pearson Correlation | -0.275 | -0.160 | -0.291 | -0.123 | -0.227 | -0.168 | 0.003 | -0.141 | -.564^**^ | -.510^*^ | -0.387 | -0.129 | 1 | .435^*^ | .409^*^ | .582^*^ | 0.388 | 0.365 | 0.032 | .437^*^ | 0.236 | 0.030 | 0.219 | .449^*^ | 0.356 | 0.154 | 0.264 | 0.217 | .556^**^ | .524^**^ | .656^**^ | -0.316 | -0.027 | -.448^*^ | -0.387 | -0.090 | -0.404 | -0.361 | -0.196 | -.518^*^ | -.480^*^ | -0.318 |
|  | Sig. (2-tailed) | 0.194 | 0.455 | 0.167 | 0.566 | 0.285 | 0.433 | 0.991 | 0.511 | 0.004 | 0.011 | 0.061 | 0.550 |  | 0.034 | 0.047 | 0.018 | 0.061 | 0.164 | 0.883 | 0.033 | 0.267 | 0.890 | 0.304 | 0.028 | 0.087 | 0.472 | 0.212 | 0.309 | 0.005 | 0.009 | 0.000 | 0.132 | 0.902 | 0.028 | 0.062 | 0.676 | 0.050 | 0.083 | 0.360 | 0.040 | 0.018 | 0.130 |
| SIRT3 | Pearson Correlation | -0.363 | -0.352 | -.542^**^ | -0.145 | -0.376 | -.433^*^ | -0.204 | -0.346 | -.453^*^ | -.694^**^ | -.587^**^ | -0.123 | .435^*^ | 1 | .672^**^ | .896^**^ | .732^**^ | 0.451 | 0.268 | .728^**^ | .618^**^ | .544^**^ | .610^**^ | .567^**^ | .676^**^ | .407^*^ | .575^**^ | .469^*^ | .638^**^ | .451^*^ | .477^*^ | -.673^**^ | -0.317 | -.509^*^ | -.448^*^ | -0.331 | -0.391 | -0.362 | -.667^**^ | -.547^*^ | -.567^**^ | -0.306 |
|  | Sig. (2-tailed) | 0.081 | 0.091 | 0.006 | 0.498 | 0.070 | 0.034 | 0.340 | 0.098 | 0.026 | 0.000 | 0.003 | 0.566 | 0.034 |  | 0.000 | 0.000 | 0.000 | 0.079 | 0.206 | 0.000 | 0.001 | 0.006 | 0.002 | 0.004 | 0.000 | 0.048 | 0.003 | 0.021 | 0.001 | 0.027 | 0.019 | 0.000 | 0.132 | 0.011 | 0.028 | 0.114 | 0.059 | 0.082 | 0.000 | 0.028 | 0.004 | 0.146 |
| CLPP | Pearson Correlation | -0.273 | -0.167 | -0.330 | 0.155 | -0.070 | -0.042 | -0.043 | -0.149 | -0.304 | -.666^**^ | -.716^**^ | 0.059 | .409^*^ | .672^**^ | 1 | .827^**^ | .766^**^ | 0.329 | 0.244 | .809^**^ | .628^**^ | .540^**^ | .507^*^ | .638^**^ | .695^**^ | .589^**^ | .657^**^ | .616^**^ | .429^*^ | .424^*^ | .620^**^ | -.653^**^ | -0.309 | -0.393 | -0.310 | -.484^*^ | -.575^**^ | -.514^*^ | -.669^**^ | -.579^*^ | -.513^*^ | -.581^**^ |
|  | Sig. (2-tailed) | 0.197 | 0.436 | 0.116 | 0.469 | 0.747 | 0.844 | 0.841 | 0.487 | 0.149 | 0.000 | 0.000 | 0.786 | 0.047 | 0.000 |  | 0.000 | 0.000 | 0.213 | 0.250 | 0.000 | 0.001 | 0.006 | 0.011 | 0.001 | 0.000 | 0.002 | 0.000 | 0.001 | 0.037 | 0.039 | 0.001 | 0.001 | 0.142 | 0.058 | 0.141 | 0.017 | 0.003 | 0.010 | 0.000 | 0.019 | 0.010 | 0.003 |
| JNK2 | Pearson Correlation | -0.213 | -0.170 | -0.301 | -0.186 | -0.172 | -0.262 | -0.009 | -0.144 | -0.384 | -.747^**^ | -.644^**^ | 0.036 | .582^*^ | .896^**^ | .827^**^ | 1 | .811^**^ | 0.343 | -0.077 | .801^**^ | .718^**^ | .774^**^ | .606^*^ | .616^*^ | .702^**^ | 0.391 | .703^**^ | 0.437 | .929^**^ | 0.349 | 0.479 | -.583^*^ | -0.433 | -0.488 | -.596^*^ | -0.185 | -.576^*^ | -0.479 | -.832^**^ | -.563^*^ | -.702^**^ | -0.483 |
|  | Sig. (2-tailed) | 0.427 | 0.529 | 0.257 | 0.491 | 0.525 | 0.328 | 0.974 | 0.596 | 0.142 | 0.001 | 0.007 | 0.896 | 0.018 | 0.000 | 0.000 |  | 0.000 | 0.193 | 0.777 | 0.000 | 0.002 | 0.000 | 0.013 | 0.011 | 0.002 | 0.134 | 0.002 | 0.090 | 0.000 | 0.186 | 0.061 | 0.018 | 0.094 | 0.055 | 0.015 | 0.492 | 0.019 | 0.060 | 0.000 | 0.023 | 0.002 | 0.058 |
| p-Jun | Pearson Correlation | -0.351 | -0.274 | -0.403 | -0.156 | -0.312 | -0.238 | -0.214 | -0.348 | -.516^**^ | -.605^**^ | -.567^**^ | -0.148 | 0.388 | .732^**^ | .766^**^ | .811^**^ | 1 | 0.475 | 0.034 | .787^**^ | .509^*^ | .634^**^ | .599^**^ | .562^**^ | .791^**^ | .449^*^ | .507^*^ | .493^*^ | .566^**^ | 0.334 | .560^**^ | -.615^**^ | -.597^**^ | -.502^*^ | -.436^*^ | -0.289 | -0.392 | -0.308 | -.594^**^ | -.541^*^ | -.624^**^ | -.464^*^ |
|  | Sig. (2-tailed) | 0.092 | 0.195 | 0.051 | 0.468 | 0.138 | 0.263 | 0.316 | 0.096 | 0.010 | 0.002 | 0.004 | 0.491 | 0.061 | 0.000 | 0.000 | 0.000 |  | 0.063 | 0.875 | 0.000 | 0.011 | 0.001 | 0.002 | 0.004 | 0.000 | 0.028 | 0.012 | 0.014 | 0.004 | 0.111 | 0.004 | 0.001 | 0.002 | 0.013 | 0.033 | 0.171 | 0.058 | 0.144 | 0.002 | 0.031 | 0.001 | 0.022 |
| HSP60 | Pearson Correlation | -.659^**^ | -0.356 | -.633^**^ | -0.420 | -.553^*^ | -0.293 | -0.274 | -.664^**^ | -.683^**^ | -0.487 | -.561^*^ | -0.407 | 0.365 | 0.451 | 0.329 | 0.343 | 0.475 | 1 | -0.215 | 0.414 | 0.200 | 0.121 | 0.364 | 0.417 | 0.444 | 0.383 | 0.177 | 0.178 | 0.299 | 0.201 | .576^*^ | -0.376 | -.664^**^ | -.628^**^ | -.579^*^ | -0.339 | -0.388 | -.546^*^ | -0.456 | -0.421 | -0.184 | -0.491 |
|  | Sig. (2-tailed) | 0.005 | 0.176 | 0.008 | 0.105 | 0.026 | 0.270 | 0.305 | 0.005 | 0.004 | 0.056 | 0.024 | 0.117 | 0.164 | 0.079 | 0.213 | 0.193 | 0.063 |  | 0.424 | 0.111 | 0.458 | 0.654 | 0.166 | 0.109 | 0.085 | 0.144 | 0.512 | 0.511 | 0.261 | 0.456 | 0.019 | 0.151 | 0.005 | 0.009 | 0.019 | 0.198 | 0.137 | 0.029 | 0.076 | 0.104 | 0.495 | 0.053 |
| eIF2a | Pearson Correlation | 0.061 | 0.007 | -0.289 | 0.218 | 0.087 | -0.007 | -0.035 | 0.018 | -0.253 | -0.240 | -0.181 | 0.011 | 0.032 | 0.268 | 0.244 | -0.077 | 0.034 | -0.215 | 1 | .424^*^ | 0.361 | -0.144 | 0.382 | .460^*^ | 0.248 | .457^*^ | .562^**^ | 0.337 | -0.163 | 0.102 | 0.256 | -0.234 | 0.135 | -0.027 | -0.303 | -0.096 | -0.223 | -0.138 | -0.233 | -0.081 | -0.127 | 0.111 |
|  | Sig. (2-tailed) | 0.778 | 0.973 | 0.170 | 0.306 | 0.687 | 0.973 | 0.870 | 0.932 | 0.233 | 0.260 | 0.398 | 0.959 | 0.883 | 0.206 | 0.250 | 0.777 | 0.875 | 0.424 |  | 0.039 | 0.083 | 0.501 | 0.066 | 0.024 | 0.242 | 0.025 | 0.004 | 0.107 | 0.445 | 0.635 | 0.228 | 0.271 | 0.529 | 0.899 | 0.150 | 0.654 | 0.296 | 0.519 | 0.274 | 0.766 | 0.556 | 0.604 |
| ATF4 | Pearson Correlation | -0.359 | -.406^*^ | -.582^**^ | -0.058 | -0.264 | -0.341 | -0.195 | -0.315 | -.588^**^ | -.760^**^ | -.756^**^ | -0.108 | .437^*^ | .728^**^ | .809^**^ | .801^**^ | .787^**^ | 0.414 | .424^*^ | 1 | .589^**^ | .501^*^ | .676^**^ | .882^**^ | .841^**^ | .799^**^ | .790^**^ | .662^**^ | .462^*^ | 0.296 | .742^**^ | -.766^**^ | -.415^*^ | -.516^**^ | -.471^*^ | -0.392 | -.615^**^ | -.498^*^ | -.680^**^ | -.614^*^ | -.591^**^ | -.494^*^ |
|  | Sig. (2-tailed) | 0.085 | 0.049 | 0.003 | 0.787 | 0.213 | 0.102 | 0.361 | 0.133 | 0.003 | 0.000 | 0.000 | 0.617 | 0.033 | 0.000 | 0.000 | 0.000 | 0.000 | 0.111 | 0.039 |  | 0.002 | 0.013 | 0.000 | 0.000 | 0.000 | 0.000 | 0.000 | 0.000 | 0.023 | 0.161 | 0.000 | 0.000 | 0.044 | 0.010 | 0.020 | 0.058 | 0.001 | 0.013 | 0.000 | 0.011 | 0.002 | 0.014 |
| CHOP | Pearson Correlation | -0.103 | -0.154 | -0.228 | -0.052 | -0.189 | -0.225 | -0.033 | -0.109 | -0.190 | -.454^*^ | -0.384 | 0.016 | 0.236 | .618^**^ | .628^**^ | .718^**^ | .509^*^ | 0.200 | 0.361 | .589^**^ | 1 | .703^**^ | .719^**^ | .532^**^ | .653^**^ | .445^*^ | .639^**^ | .505^*^ | .488^*^ | .458^*^ | .464^*^ | -0.342 | -0.185 | -0.265 | -.430^*^ | -.495^*^ | -0.383 | -0.381 | -.669^**^ | -.537^*^ | -.443^*^ | -.632^**^ |
|  | Sig. (2-tailed) | 0.633 | 0.473 | 0.285 | 0.810 | 0.377 | 0.291 | 0.879 | 0.611 | 0.375 | 0.026 | 0.064 | 0.941 | 0.267 | 0.001 | 0.001 | 0.002 | 0.011 | 0.458 | 0.083 | 0.002 |  | 0.000 | 0.000 | 0.007 | 0.001 | 0.029 | 0.001 | 0.012 | 0.016 | 0.024 | 0.022 | 0.101 | 0.386 | 0.211 | 0.036 | 0.014 | 0.065 | 0.066 | 0.000 | 0.032 | 0.030 | 0.001 |
| IRE1 | Pearson Correlation | -0.151 | -0.205 | -0.151 | -0.166 | -0.255 | -0.267 | -0.116 | -0.146 | -0.102 | -0.359 | -0.376 | -0.007 | 0.030 | .544^**^ | .540^**^ | .774^**^ | .634^**^ | 0.121 | -0.144 | .501^*^ | .703^**^ | 1 | .568^**^ | 0.372 | .557^**^ | 0.323 | .408^*^ | .424^*^ | .600^**^ | 0.210 | 0.235 | -0.400 | -0.368 | -0.200 | -0.273 | -0.253 | -0.172 | -0.200 | -.631^**^ | -.571^*^ | -.510^*^ | -.518^**^ |
|  | Sig. (2-tailed) | 0.482 | 0.336 | 0.482 | 0.437 | 0.229 | 0.207 | 0.588 | 0.496 | 0.635 | 0.085 | 0.070 | 0.973 | 0.890 | 0.006 | 0.006 | 0.000 | 0.001 | 0.654 | 0.501 | 0.013 | 0.000 |  | 0.004 | 0.073 | 0.005 | 0.124 | 0.048 | 0.039 | 0.002 | 0.324 | 0.268 | 0.053 | 0.077 | 0.350 | 0.197 | 0.234 | 0.420 | 0.350 | 0.001 | 0.021 | 0.011 | 0.009 |
| XBP1 | Pearson Correlation | -0.295 | -0.321 | -.512^*^ | -0.385 | -.470^*^ | -0.384 | -0.333 | -.491^*^ | -.479^*^ | -.615^**^ | -.510^*^ | -0.365 | 0.219 | .610^**^ | .507^*^ | .606^*^ | .599^**^ | 0.364 | 0.382 | .676^**^ | .719^**^ | .568^**^ | 1 | .616^**^ | .719^**^ | .593^**^ | .594^**^ | .433^*^ | 0.404 | 0.214 | .519^**^ | -.466^*^ | -.544^**^ | -.576^**^ | -.558^**^ | -.441^*^ | -.541^**^ | -0.358 | -.621^**^ | -.682^**^ | -.449^*^ | -.431^*^ |
|  | Sig. (2-tailed) | 0.161 | 0.127 | 0.011 | 0.063 | 0.021 | 0.064 | 0.112 | 0.015 | 0.018 | 0.001 | 0.011 | 0.080 | 0.304 | 0.002 | 0.011 | 0.013 | 0.002 | 0.166 | 0.066 | 0.000 | 0.000 | 0.004 |  | 0.001 | 0.000 | 0.002 | 0.002 | 0.034 | 0.050 | 0.315 | 0.009 | 0.022 | 0.006 | 0.003 | 0.005 | 0.031 | 0.006 | 0.086 | 0.001 | 0.004 | 0.028 | 0.036 |
| GRP78 | Pearson Correlation | -0.399 | -0.355 | -.588^**^ | -0.167 | -0.231 | -0.361 | -0.275 | -0.373 | -.632^**^ | -.772^**^ | -.721^**^ | -0.209 | .449^*^ | .567^**^ | .638^**^ | .616^*^ | .562^**^ | 0.417 | .460^*^ | .882^**^ | .532^**^ | 0.372 | .616^**^ | 1 | .749^**^ | .845^**^ | .852^**^ | .691^**^ | 0.393 | 0.342 | .729^**^ | -.728^**^ | -0.345 | -.460^*^ | -.561^**^ | -0.298 | -.619^**^ | -.462^*^ | -.574^**^ | -.689^**^ | -.671^**^ | -.452^*^ |
|  | Sig. (2-tailed) | 0.053 | 0.089 | 0.002 | 0.434 | 0.278 | 0.083 | 0.193 | 0.072 | 0.001 | 0.000 | 0.000 | 0.327 | 0.028 | 0.004 | 0.001 | 0.011 | 0.004 | 0.109 | 0.024 | 0.000 | 0.007 | 0.073 | 0.001 |  | 0.000 | 0.000 | 0.000 | 0.000 | 0.058 | 0.102 | 0.000 | 0.000 | 0.098 | 0.024 | 0.004 | 0.158 | 0.001 | 0.023 | 0.003 | 0.003 | 0.000 | 0.027 |
| UBA1 | Pearson Correlation | -.567^**^ | -.582^**^ | -.678^**^ | -.417^*^ | -.510^*^ | -.541^**^ | -.451^*^ | -.540^**^ | -.603^**^ | -.826^**^ | -.687^**^ | -0.384 | 0.356 | .676^**^ | .695^**^ | .702^**^ | .791^**^ | 0.444 | 0.248 | .841^**^ | .653^**^ | .557^**^ | .719^**^ | .749^**^ | 1 | .620^**^ | .704^**^ | .641^**^ | .579^**^ | 0.369 | .666^**^ | -.687^**^ | -.551^**^ | -.593^**^ | -.641^**^ | -.622^**^ | -.612^**^ | -0.309 | -.626^**^ | -0.465 | -.630^**^ | -.541^**^ |
|  | Sig. (2-tailed) | 0.004 | 0.003 | 0.000 | 0.043 | 0.011 | 0.006 | 0.027 | 0.006 | 0.002 | 0.000 | 0.000 | 0.064 | 0.087 | 0.000 | 0.000 | 0.002 | 0.000 | 0.085 | 0.242 | 0.000 | 0.001 | 0.005 | 0.000 | 0.000 |  | 0.001 | 0.000 | 0.001 | 0.003 | 0.076 | 0.000 | 0.000 | 0.005 | 0.002 | 0.001 | 0.001 | 0.001 | 0.142 | 0.001 | 0.069 | 0.001 | 0.006 |
| UBA2 | Pearson Correlation | -0.397 | -0.373 | -.568^**^ | -0.105 | -0.206 | -0.368 | -0.388 | -0.393 | -.452^*^ | -.612^**^ | -.590^**^ | -0.243 | 0.154 | .407^*^ | .589^**^ | 0.391 | .449^*^ | 0.383 | .457^*^ | .799^**^ | .445^*^ | 0.323 | .593^**^ | .845^**^ | .620^**^ | 1 | .771^**^ | .716^**^ | 0.135 | 0.183 | .496^*^ | -.609^**^ | -0.331 | -0.402 | -0.384 | -.420^*^ | -.577^**^ | -.507^*^ | -.601^**^ | -.579^*^ | -0.358 | -.431^*^ |
|  | Sig. (2-tailed) | 0.055 | 0.073 | 0.004 | 0.626 | 0.335 | 0.077 | 0.061 | 0.057 | 0.027 | 0.001 | 0.002 | 0.252 | 0.472 | 0.048 | 0.002 | 0.134 | 0.028 | 0.144 | 0.025 | 0.000 | 0.029 | 0.124 | 0.002 | 0.000 | 0.001 |  | 0.000 | 0.000 | 0.528 | 0.391 | 0.014 | 0.002 | 0.115 | 0.051 | 0.064 | 0.041 | 0.003 | 0.012 | 0.002 | 0.019 | 0.086 | 0.036 |
| UBE2B | Pearson Correlation | -0.325 | -0.275 | -.499^*^ | 0.005 | -0.288 | -0.311 | -0.319 | -0.277 | -.483^*^ | -.618^**^ | -.564^**^ | -0.216 | 0.264 | .575^**^ | .657^**^ | .703^**^ | .507^*^ | 0.177 | .562^**^ | .790^**^ | .639^**^ | .408^*^ | .594^**^ | .852^**^ | .704^**^ | .771^**^ | 1 | .844^**^ | 0.257 | .440^*^ | .552^**^ | -.595^**^ | -0.167 | -0.296 | -.459^*^ | -.457^*^ | -.473^*^ | -.418^*^ | -.676^**^ | -.601^*^ | -.511^*^ | -.467^*^ |
|  | Sig. (2-tailed) | 0.121 | 0.193 | 0.013 | 0.980 | 0.172 | 0.139 | 0.128 | 0.190 | 0.017 | 0.001 | 0.004 | 0.310 | 0.212 | 0.003 | 0.000 | 0.002 | 0.012 | 0.512 | 0.004 | 0.000 | 0.001 | 0.048 | 0.002 | 0.000 | 0.000 | 0.000 |  | 0.000 | 0.225 | 0.032 | 0.005 | 0.002 | 0.435 | 0.160 | 0.024 | 0.025 | 0.020 | 0.042 | 0.000 | 0.014 | 0.011 | 0.021 |
| UBE2K | Pearson Correlation | -.537^**^ | -0.248 | -.516^**^ | -0.035 | -0.326 | -.410^*^ | -.453^*^ | -0.349 | -.478^*^ | -.570^**^ | -.543^**^ | -0.299 | 0.217 | .469^*^ | .616^**^ | 0.437 | .493^*^ | 0.178 | 0.337 | .662^**^ | .505^*^ | .424^*^ | .433^*^ | .691^**^ | .641^**^ | .716^**^ | .844^**^ | 1 | 0.228 | .474^*^ | .498^*^ | -.525^**^ | -0.210 | -0.166 | -0.360 | -.474^*^ | -0.266 | -0.181 | -.702^**^ | -0.473 | -.522^**^ | -.461^*^ |
|  | Sig. (2-tailed) | 0.007 | 0.242 | 0.010 | 0.871 | 0.121 | 0.046 | 0.026 | 0.095 | 0.018 | 0.004 | 0.006 | 0.156 | 0.309 | 0.021 | 0.001 | 0.090 | 0.014 | 0.511 | 0.107 | 0.000 | 0.012 | 0.039 | 0.034 | 0.000 | 0.001 | 0.000 | 0.000 |  | 0.284 | 0.019 | 0.013 | 0.008 | 0.324 | 0.439 | 0.084 | 0.019 | 0.209 | 0.397 | 0.000 | 0.064 | 0.009 | 0.023 |
| UBE3 | Pearson Correlation | -0.323 | -.441^*^ | -0.400 | -.425^*^ | -0.399 | -.468^*^ | -0.157 | -0.258 | -0.387 | -.587^**^ | -.475^*^ | -0.176 | .556^**^ | .638^**^ | .429^*^ | .929^**^ | .566^**^ | 0.299 | -0.163 | .462^*^ | .488^*^ | .600^**^ | 0.404 | 0.393 | .579^**^ | 0.135 | 0.257 | 0.228 | 1 | 0.367 | 0.403 | -0.364 | -0.157 | -0.343 | -.586^**^ | -0.193 | -0.271 | -0.101 | -.475^*^ | -.553^*^ | -.617^**^ | -0.392 |
|  | Sig. (2-tailed) | 0.124 | 0.031 | 0.053 | 0.038 | 0.054 | 0.021 | 0.465 | 0.223 | 0.062 | 0.003 | 0.019 | 0.410 | 0.005 | 0.001 | 0.037 | 0.000 | 0.004 | 0.261 | 0.445 | 0.023 | 0.016 | 0.002 | 0.050 | 0.058 | 0.003 | 0.528 | 0.225 | 0.284 |  | 0.078 | 0.051 | 0.081 | 0.463 | 0.101 | 0.003 | 0.366 | 0.200 | 0.639 | 0.019 | 0.026 | 0.001 | 0.058 |
| ATGL | Pearson Correlation | -0.307 | -0.104 | -0.220 | -0.011 | -0.137 | -0.121 | -0.135 | -0.141 | -0.380 | -0.336 | -0.264 | -0.077 | .524^**^ | .451^*^ | .424^*^ | 0.349 | 0.334 | 0.201 | 0.102 | 0.296 | .458^*^ | 0.210 | 0.214 | 0.342 | 0.369 | 0.183 | .440^*^ | .474^*^ | 0.367 | 1 | 0.314 | -0.246 | -0.037 | -0.213 | -0.254 | -0.256 | -0.052 | -0.016 | -0.338 | -0.472 | -.493^*^ | -0.249 |
|  | Sig. (2-tailed) | 0.144 | 0.629 | 0.301 | 0.958 | 0.524 | 0.574 | 0.529 | 0.512 | 0.067 | 0.109 | 0.213 | 0.721 | 0.009 | 0.027 | 0.039 | 0.186 | 0.111 | 0.456 | 0.635 | 0.161 | 0.024 | 0.324 | 0.315 | 0.102 | 0.076 | 0.391 | 0.032 | 0.019 | 0.078 |  | 0.135 | 0.247 | 0.863 | 0.319 | 0.231 | 0.227 | 0.809 | 0.941 | 0.107 | 0.065 | 0.014 | 0.240 |
| p-AMPK | Pearson Correlation | -0.373 | -0.236 | -.512^*^ | -0.069 | -0.293 | -0.222 | 0.001 | -0.328 | -.614^**^ | -.652^**^ | -.732^**^ | -0.097 | .656^**^ | .477^*^ | .620^**^ | 0.479 | .560^**^ | .576^*^ | 0.256 | .742^**^ | .464^*^ | 0.235 | .519^**^ | .729^**^ | .666^**^ | .496^*^ | .552^**^ | .498^*^ | 0.403 | 0.314 | 1 | -.571^**^ | -0.335 | -.416^*^ | -.473^*^ | -0.365 | -.530^**^ | -.437^*^ | -.503^*^ | -.566^*^ | -.499^*^ | -.611^**^ |
|  | Sig. (2-tailed) | 0.073 | 0.266 | 0.011 | 0.747 | 0.164 | 0.297 | 0.995 | 0.117 | 0.001 | 0.001 | 0.000 | 0.652 | 0.000 | 0.019 | 0.001 | 0.061 | 0.004 | 0.019 | 0.228 | 0.000 | 0.022 | 0.268 | 0.009 | 0.000 | 0.000 | 0.014 | 0.005 | 0.013 | 0.051 | 0.135 |  | 0.004 | 0.110 | 0.043 | 0.020 | 0.079 | 0.008 | 0.033 | 0.012 | 0.022 | 0.013 | 0.002 |
| GSH-Px | Pearson Correlation | .425^*^ | .444^*^ | .584^**^ | 0.223 | 0.329 | .417^*^ | 0.310 | .439^*^ | .448^*^ | .805^**^ | .698^**^ | 0.148 | -0.316 | -.673^**^ | -.653^**^ | -.583^*^ | -.615^**^ | -0.376 | -0.234 | -.766^**^ | -0.342 | -0.400 | -.466^*^ | -.728^**^ | -.687^**^ | -.609^**^ | -.595^**^ | -.525^**^ | -0.364 | -0.246 | -.571^**^ | 1 | 0.331 | .587^**^ | .441^*^ | 0.393 | .612^**^ | 0.366 | .418^*^ | .705^**^ | .604^**^ | 0.276 |
|  | Sig. (2-tailed) | 0.039 | 0.030 | 0.003 | 0.295 | 0.116 | 0.043 | 0.141 | 0.032 | 0.028 | 0.000 | 0.000 | 0.489 | 0.132 | 0.000 | 0.001 | 0.018 | 0.001 | 0.151 | 0.271 | 0.000 | 0.101 | 0.053 | 0.022 | 0.000 | 0.000 | 0.002 | 0.002 | 0.008 | 0.081 | 0.247 | 0.004 |  | 0.114 | 0.003 | 0.031 | 0.057 | 0.001 | 0.079 | 0.042 | 0.002 | 0.002 | 0.192 |
| T-AOC | Pearson Correlation | .466^*^ | 0.233 | 0.403 | .457^*^ | 0.290 | 0.317 | 0.373 | .509^*^ | .499^*^ | .444^*^ | .444^*^ | 0.375 | -0.027 | -0.317 | -0.309 | -0.433 | -.597^**^ | -.664^**^ | 0.135 | -.415^*^ | -0.185 | -0.368 | -.544^**^ | -0.345 | -.551^**^ | -0.331 | -0.167 | -0.210 | -0.157 | -0.037 | -0.335 | 0.331 | 1 | .560^**^ | 0.296 | 0.223 | 0.354 | 0.120 | 0.197 | 0.340 | 0.401 | 0.216 |
|  | Sig. (2-tailed) | 0.022 | 0.273 | 0.051 | 0.025 | 0.169 | 0.131 | 0.073 | 0.011 | 0.013 | 0.030 | 0.030 | 0.071 | 0.902 | 0.132 | 0.142 | 0.094 | 0.002 | 0.005 | 0.529 | 0.044 | 0.386 | 0.077 | 0.006 | 0.098 | 0.005 | 0.115 | 0.435 | 0.324 | 0.463 | 0.863 | 0.110 | 0.114 |  | 0.004 | 0.160 | 0.294 | 0.089 | 0.577 | 0.355 | 0.197 | 0.052 | 0.312 |
| T-SOD | Pearson Correlation | .513^*^ | .633^**^ | .583^**^ | .597^**^ | .576^**^ | .508^*^ | .457^*^ | .597^**^ | .580^**^ | .697^**^ | .509^*^ | .515^*^ | -.448^*^ | -.509^*^ | -0.393 | -0.488 | -.502^*^ | -.628^**^ | -0.027 | -.516^**^ | -0.265 | -0.200 | -.576^**^ | -.460^*^ | -.593^**^ | -0.402 | -0.296 | -0.166 | -0.343 | -0.213 | -.416^*^ | .587^**^ | .560^**^ | 1 | .443^*^ | .533^**^ | .745^**^ | .457^*^ | 0.096 | .520^*^ | 0.323 | 0.307 |
|  | Sig. (2-tailed) | 0.010 | 0.001 | 0.003 | 0.002 | 0.003 | 0.011 | 0.025 | 0.002 | 0.003 | 0.000 | 0.011 | 0.010 | 0.028 | 0.011 | 0.058 | 0.055 | 0.013 | 0.009 | 0.899 | 0.010 | 0.211 | 0.350 | 0.003 | 0.024 | 0.002 | 0.051 | 0.160 | 0.439 | 0.101 | 0.319 | 0.043 | 0.003 | 0.004 |  | 0.030 | 0.007 | 0.000 | 0.025 | 0.654 | 0.039 | 0.124 | 0.145 |
| ATP | Pearson Correlation | .475^*^ | 0.367 | .619^**^ | .604^**^ | .549^**^ | .537^**^ | .524^**^ | .509^*^ | .609^**^ | .649^**^ | 0.401 | .556^**^ | -0.387 | -.448^*^ | -0.310 | -.596^*^ | -.436^*^ | -.579^*^ | -0.303 | -.471^*^ | -.430^*^ | -0.273 | -.558^**^ | -.561^**^ | -.641^**^ | -0.384 | -.459^*^ | -0.360 | -.586^**^ | -0.254 | -.473^*^ | .441^*^ | 0.296 | .443^*^ | 1 | 0.338 | .457^*^ | 0.168 | 0.352 | .632^**^ | .593^**^ | 0.243 |
|  | Sig. (2-tailed) | 0.019 | 0.077 | 0.001 | 0.002 | 0.005 | 0.007 | 0.009 | 0.011 | 0.002 | 0.001 | 0.052 | 0.005 | 0.062 | 0.028 | 0.141 | 0.015 | 0.033 | 0.019 | 0.150 | 0.020 | 0.036 | 0.197 | 0.005 | 0.004 | 0.001 | 0.064 | 0.024 | 0.084 | 0.003 | 0.231 | 0.020 | 0.031 | 0.160 | 0.030 |  | 0.106 | 0.025 | 0.434 | 0.092 | 0.009 | 0.002 | 0.253 |
| SOD2 | Pearson Correlation | .519^**^ | .589^**^ | .526^**^ | 0.342 | .574^**^ | .489^*^ | .449^*^ | .487^*^ | 0.189 | .547^**^ | .442^*^ | .415^*^ | -0.090 | -0.331 | -.484^*^ | -0.185 | -0.289 | -0.339 | -0.096 | -0.392 | -.495^*^ | -0.253 | -.441^*^ | -0.298 | -.622^**^ | -.420^*^ | -.457^*^ | -.474^*^ | -0.193 | -0.256 | -0.365 | 0.393 | 0.223 | .533^**^ | 0.338 | 1 | .605^**^ | .412^*^ | .407^*^ | 0.084 | 0.009 | .612^**^ |
|  | Sig. (2-tailed) | 0.009 | 0.002 | 0.008 | 0.102 | 0.003 | 0.015 | 0.028 | 0.016 | 0.375 | 0.006 | 0.030 | 0.044 | 0.676 | 0.114 | 0.017 | 0.492 | 0.171 | 0.198 | 0.654 | 0.058 | 0.014 | 0.234 | 0.031 | 0.158 | 0.001 | 0.041 | 0.025 | 0.019 | 0.366 | 0.227 | 0.079 | 0.057 | 0.294 | 0.007 | 0.106 |  | 0.002 | 0.045 | 0.048 | 0.757 | 0.966 | 0.001 |
| ACACA | Pearson Correlation | 0.257 | 0.402 | .416^*^ | 0.335 | 0.263 | 0.349 | 0.223 | 0.301 | 0.294 | .744^**^ | .529^**^ | 0.233 | -0.404 | -0.391 | -.575^**^ | -.576^*^ | -0.392 | -0.388 | -0.223 | -.615^**^ | -0.383 | -0.172 | -.541^**^ | -.619^**^ | -.612^**^ | -.577^**^ | -.473^*^ | -0.266 | -0.271 | -0.052 | -.530^**^ | .612^**^ | 0.354 | .745^**^ | .457^*^ | .605^**^ | 1 | .664^**^ | 0.217 | 0.432 | 0.279 | .434^*^ |
|  | Sig. (2-tailed) | 0.225 | 0.051 | 0.043 | 0.110 | 0.214 | 0.095 | 0.294 | 0.154 | 0.164 | 0.000 | 0.008 | 0.273 | 0.050 | 0.059 | 0.003 | 0.019 | 0.058 | 0.137 | 0.296 | 0.001 | 0.065 | 0.420 | 0.006 | 0.001 | 0.001 | 0.003 | 0.020 | 0.209 | 0.200 | 0.809 | 0.008 | 0.001 | 0.089 | 0.000 | 0.025 | 0.002 |  | 0.000 | 0.308 | 0.095 | 0.187 | 0.034 |
| FASN | Pearson Correlation | 0.110 | 0.173 | 0.225 | -0.082 | 0.188 | 0.036 | 0.010 | 0.174 | 0.109 | 0.352 | 0.324 | 0.047 | -0.361 | -0.362 | -.514^*^ | -0.479 | -0.308 | -.546^*^ | -0.138 | -.498^*^ | -0.381 | -0.200 | -0.358 | -.462^*^ | -0.309 | -.507^*^ | -.418^*^ | -0.181 | -0.101 | -0.016 | -.437^*^ | 0.366 | 0.120 | .457^*^ | 0.168 | .412^*^ | .664^**^ | 1 | 0.385 | 0.334 | -0.061 | .572^**^ |
|  | Sig. (2-tailed) | 0.610 | 0.419 | 0.290 | 0.703 | 0.378 | 0.866 | 0.964 | 0.415 | 0.612 | 0.092 | 0.122 | 0.827 | 0.083 | 0.082 | 0.010 | 0.060 | 0.144 | 0.029 | 0.519 | 0.013 | 0.066 | 0.350 | 0.086 | 0.023 | 0.142 | 0.012 | 0.042 | 0.397 | 0.639 | 0.941 | 0.033 | 0.079 | 0.577 | 0.025 | 0.434 | 0.045 | 0.000 |  | 0.063 | 0.206 | 0.777 | 0.003 |
| p-mTOR | Pearson Correlation | 0.382 | 0.210 | .450^*^ | -0.034 | 0.348 | 0.246 | 0.224 | 0.349 | 0.256 | .493^*^ | .554^**^ | 0.165 | -0.196 | -.667^**^ | -.669^**^ | -.832^**^ | -.594^**^ | -0.456 | -0.233 | -.680^**^ | -.669^**^ | -.631^**^ | -.621^**^ | -.574^**^ | -.626^**^ | -.601^**^ | -.676^**^ | -.702^**^ | -.475^*^ | -0.338 | -.503^*^ | .418^*^ | 0.197 | 0.096 | 0.352 | .407^*^ | 0.217 | 0.385 | 1 | .638^**^ | 0.369 | .557^**^ |
|  | Sig. (2-tailed) | 0.065 | 0.326 | 0.027 | 0.876 | 0.096 | 0.246 | 0.292 | 0.094 | 0.227 | 0.014 | 0.005 | 0.440 | 0.360 | 0.000 | 0.000 | 0.000 | 0.002 | 0.076 | 0.274 | 0.000 | 0.000 | 0.001 | 0.001 | 0.003 | 0.001 | 0.002 | 0.000 | 0.000 | 0.019 | 0.107 | 0.012 | 0.042 | 0.355 | 0.654 | 0.092 | 0.048 | 0.308 | 0.063 |  | 0.008 | 0.076 | 0.005 |
| p-AKT | Pearson Correlation | 0.291 | 0.117 | 0.376 | 0.286 | 0.278 | 0.120 | 0.121 | 0.405 | .513^*^ | .629^**^ | .595^*^ | 0.139 | -.518^*^ | -.547^*^ | -.579^*^ | -.563^*^ | -.541^*^ | -0.421 | -0.081 | -.614^*^ | -.537^*^ | -.571^*^ | -.682^**^ | -.689^**^ | -0.465 | -.579^*^ | -.601^*^ | -0.473 | -.553^*^ | -0.472 | -.566^*^ | .705^**^ | 0.340 | .520^*^ | .632^**^ | 0.084 | 0.432 | 0.334 | .638^**^ | 1 | .667^**^ | 0.368 |
|  | Sig. (2-tailed) | 0.273 | 0.667 | 0.152 | 0.283 | 0.297 | 0.658 | 0.656 | 0.120 | 0.042 | 0.009 | 0.015 | 0.607 | 0.040 | 0.028 | 0.019 | 0.023 | 0.031 | 0.104 | 0.766 | 0.011 | 0.032 | 0.021 | 0.004 | 0.003 | 0.069 | 0.019 | 0.014 | 0.064 | 0.026 | 0.065 | 0.022 | 0.002 | 0.197 | 0.039 | 0.009 | 0.757 | 0.095 | 0.206 | 0.008 |  | 0.005 | 0.161 |
| p-S6K1 | Pearson Correlation | 0.373 | 0.121 | 0.344 | 0.318 | 0.146 | 0.299 | 0.273 | 0.262 | .615^**^ | .691^**^ | .570^**^ | 0.165 | -.480^*^ | -.567^**^ | -.513^*^ | -.702^**^ | -.624^**^ | -0.184 | -0.127 | -.591^**^ | -.443^*^ | -.510^*^ | -.449^*^ | -.671^**^ | -.630^**^ | -0.358 | -.511^*^ | -.522^**^ | -.617^**^ | -.493^*^ | -.499^*^ | .604^**^ | 0.401 | 0.323 | .593^**^ | 0.009 | 0.279 | -0.061 | 0.369 | .667^**^ | 1 | 0.218 |
|  | Sig. (2-tailed) | 0.072 | 0.574 | 0.100 | 0.130 | 0.497 | 0.157 | 0.197 | 0.217 | 0.001 | 0.000 | 0.004 | 0.440 | 0.018 | 0.004 | 0.010 | 0.002 | 0.001 | 0.495 | 0.556 | 0.002 | 0.030 | 0.011 | 0.028 | 0.000 | 0.001 | 0.086 | 0.011 | 0.009 | 0.001 | 0.014 | 0.013 | 0.002 | 0.052 | 0.124 | 0.002 | 0.966 | 0.187 | 0.777 | 0.076 | 0.005 |  | 0.306 |
| SREBP1 | Pearson Correlation | 0.302 | 0.295 | 0.257 | 0.103 | 0.318 | 0.177 | 0.068 | 0.286 | 0.251 | .423^*^ | .541^**^ | 0.075 | -0.318 | -0.306 | -.581^**^ | -0.483 | -.464^*^ | -0.491 | 0.111 | -.494^*^ | -.632^**^ | -.518^**^ | -.431^*^ | -.452^*^ | -.541^**^ | -.431^*^ | -.467^*^ | -.461^*^ | -0.392 | -0.249 | -.611^**^ | 0.276 | 0.216 | 0.307 | 0.243 | .612^**^ | .434^*^ | .572^**^ | .557^**^ | 0.368 | 0.218 | 1 |
|  | Sig. (2-tailed) | 0.152 | 0.162 | 0.225 | 0.632 | 0.130 | 0.407 | 0.751 | 0.175 | 0.237 | 0.039 | 0.006 | 0.728 | 0.130 | 0.146 | 0.003 | 0.058 | 0.022 | 0.053 | 0.604 | 0.014 | 0.001 | 0.009 | 0.036 | 0.027 | 0.006 | 0.036 | 0.021 | 0.023 | 0.058 | 0.240 | 0.002 | 0.192 | 0.312 | 0.145 | 0.253 | 0.001 | 0.034 | 0.003 | 0.005 | 0.161 | 0.306 |  |
| ^**^Correlation is significant at the 0.01 level (2-tailed) | | | | | | | | | | | | | | | | | | | | | | | | | | | | | | | | | | | | | | | | | | | |
| ^*^Correlation is significant at the 0.05 level (2-tailed) | | | | | | | | | | | | | | | | | | | | | | | | | | | | | | | | | | | | | | | | | | | |
