# Supplementary figures and images for: Selenoproteins synergistically protect porcine skeletal muscle from oxidative damage via relieving mitochondrial dysfunction and endoplasmic reticulum stress
Source: J Anim Sci Biotechnol. 2023 Jun 4;14:79. doi: 10.1186/s40104-023-00877-6 (PMC10239589; doi:10.1186/s40104-023-00877-6)

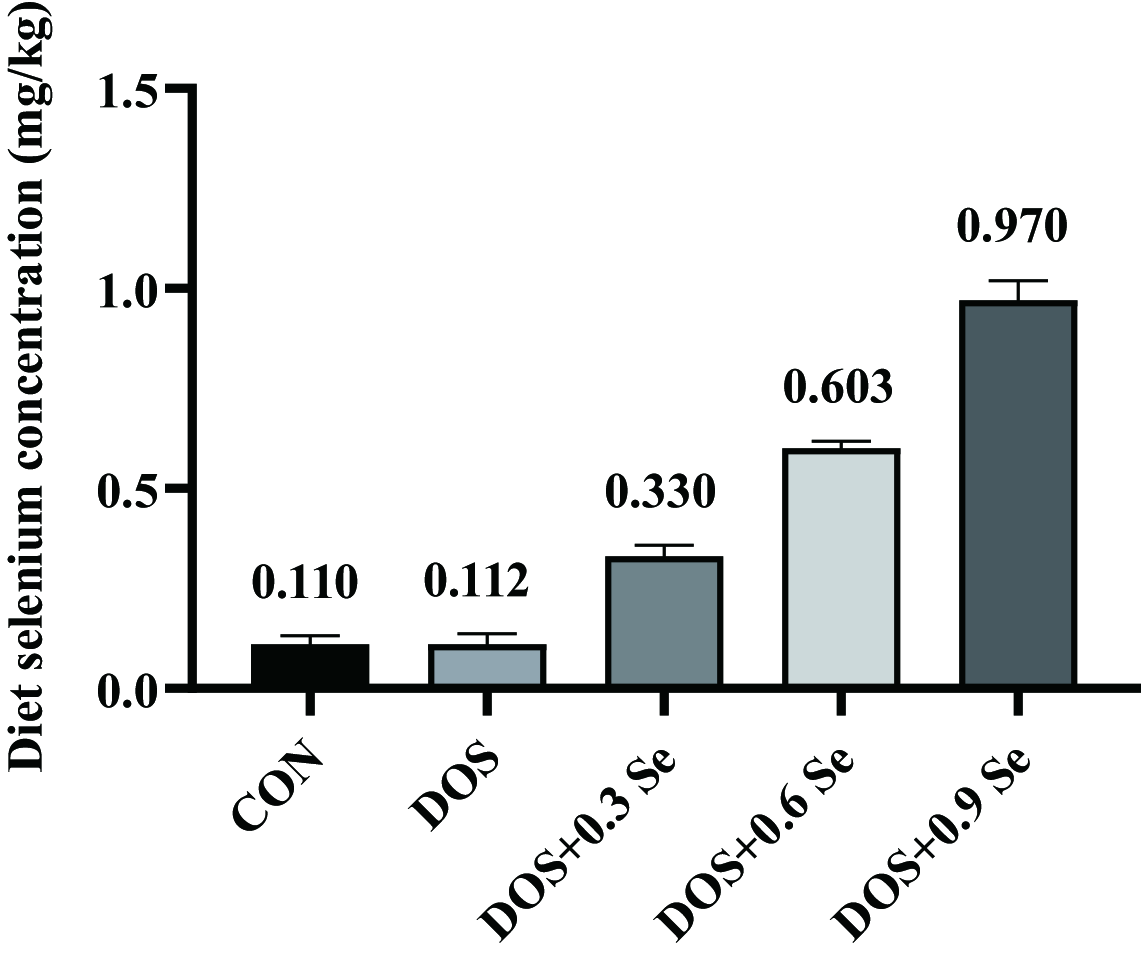

Supplement: Supplementary file 2 — Additional file 2: Fig. S1. Diet Se concentration. [file 40104_2023_877_MOESM2_ESM.tif]

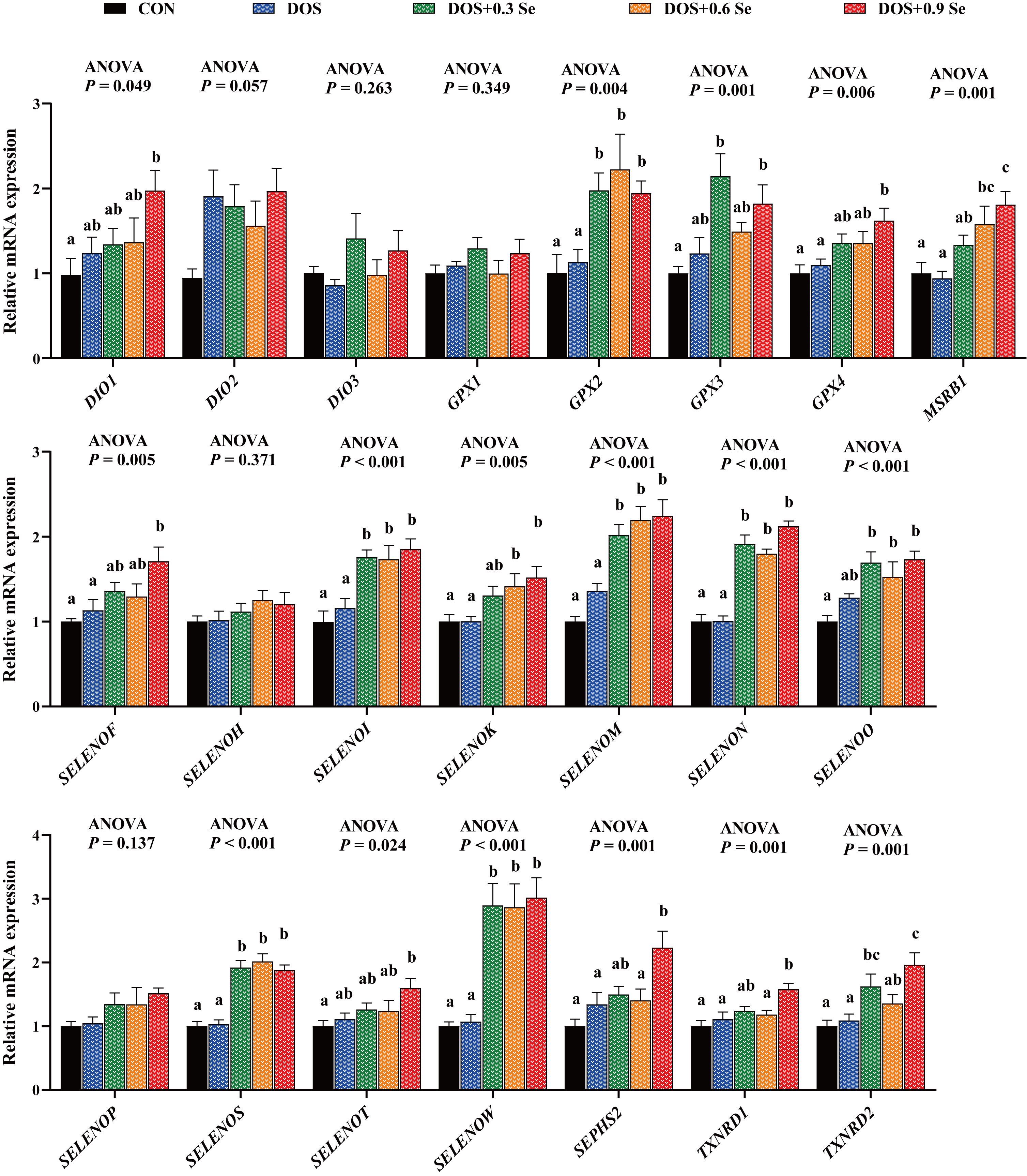

Supplement: Supplementary file 3 — Additional file 3: Fig. S2. The mRNA expression of the 22 selenogenes. [file 40104_2023_877_MOESM3_ESM.tif]
